# Textual emotion recognition to improve real-time communication of disabled people in sustainable environments using an ensemble deep learning approach

**DOI:** 10.1038/s41598-025-25363-z

**Published:** 2025-11-21

**Authors:** Turki Ali Alghamdi, Saud S. Alotaibi, Reem Alharthi

**Affiliations:** 1 Department of Computer Science and Artificial Intelligence, College of Computing, Umm Al-Qura University, Mecca, Saudi Arabia; 2 Applied College, University of Hafr Albatin, 39524 Hafr Albatin, Saudi Arabia; 3 King Salman Centre for Disability Research, 11614 Riyadh, Saudi Arabia

**Keywords:** Textual emotion recognition, Ensemble models, Improved sand cat swarm optimization, Disabled people, Text pre-processing, Computational biology and bioinformatics, Mathematics and computing

## Abstract

Social media platforms are prevalently used to express and share opinions on a wide range of topics, which has amplified interest in textual emotion detection. However, accurately detecting emotions in individuals, especially those with communication challenges, remains a complex task. Emotion analysis serves as a significant tool for assessing, monitoring, and interpreting a user’s sentiments toward services or products. The emergence of deep learning (DL) has significantly advanced this field, allowing the development of more accurate and robust models. DL techniques, particularly neural networks, have demonstrated superior performance in recognizing emotions from text, presenting enhanced capabilities for real-time sentiment understanding and user experience improvement. This manuscript presents an Optimised Ensemble Model for Precise Textual Emotion Recognition Using an Improved Sand Cat Swarm Optimization (OEMPTER-ISCSO) method. The primary objective of the OEMPTER-ISCSO method is to accurately recognize emotions in text, facilitating enhanced communication with individuals with disabilities. Initially, the text pre-processing stage involves multiple levels to normalize and clean the input text. Furthermore, the FastText method is employed for the word embedding process, transforming words into numerical vector representations. For textual emotion detection, an ensemble of three classifiers, such as the enhanced deep belief network (EDBN), Elman neural network (ELNN), and an improved temporal convolutional network (ITCN) method, is employed. Finally, the enhanced sand cat swarm optimization (ISCO) method-based hyperparameter selection procedure is executed to optimize the detection outcomes of the ensemble models. The OEMPTER-ISCSO technique achieved a superior accuracy of 95.84% in a comparative analysis on a text-based emotion detection dataset, demonstrating its efficiency over existing models.

## Introduction

Emotional investigation is crucial for the development of affective interfaces that provide suitable emotional responses, thereby creating a sense of emotional engagement and facilitating online communication^1^. Emotion recognition plays a significant role in several fields of life, specifically in driver assistance systems (DAS) and active and assisted living (AAL). Emotion Recognition is one of the technical AAL enablers, as it is deliberated to provide substantial assistance in observing and monitoring the mental state of elderly and disabled persons^2^. Additionally, it is evident from advanced publications that the classification performance of emotion recognition methods is steadily improving, and the opportunities for automated emotion recognition methods have also expanded. Multiple model types are utilized to identify emotions in humans, including body movements, facial expressions, textual information, heartbeat, and blood pressure^3^. In computational linguistics, human emotion detection utilizing text becomes more important from an application perspective. Currently, there is a massive amount of textual data on the internet. Textual emotion recognition is a computational experiment that analyses natural language in text to identify its combinations with emotions, such as fear, anger, sadness, joy, and others^4^. It is possible in various government organizations, industries, and media applications. Textual emotional detection aims to identify the primary emotion affected by analyzing their input texts^5^.

It depends on the assumption that if a person is happy, it controls them to utilize positive words. In business development, emotion detection can help marketers develop effective approaches for new product development, service delivery, and customer relationship management (CRM)^6^. Psychologists can infer an individual’s emotions based on the text they write and use to predict their mental state^7^. This knowledge might be practically employed to predict customer preferences and user behaviour for corporate economic gain. Text-based emotion detection might be utilized in psychology, education, business, and other fields. In the past decade, emotion detection from text has been explored to identify users’ emotional states from multimodal resources, including gestures, eye gazes, and audio^8^. Technology utilizing emotion detection models can automatically create emotions. One specific field that stands to benefit from dependable emotion detection methods is artificial intelligence (AI)^9^. AI software equipped with effective emotion recognition might be employed to enhance effective human-computer interaction devices. Preceding investigations on affective computing employed approaches from classical machine learning (ML), unlike the latest DL field advances^10^. ML and DL-based methods are used to calculate emotions, which have more significant results than traditional ones.

This manuscript presents an Optimised Ensemble Model for Precise Textual Emotion Recognition Using an Improved Sand Cat Swarm Optimization (OEMPTER-ISCSO) method. The primary objective of the OEMPTER-ISCSO method is to accurately recognize emotions in text, facilitating enhanced communication with individuals with disabilities. Initially, the text pre-processing stage involves multiple levels to normalize and clean the input text. Furthermore, the FastText method is employed for the word embedding process, transforming words into numerical vector representations. For textual emotion detection, an ensemble of three classifiers, such as the enhanced deep belief network (EDBN), Elman neural network (ELNN), and an improved temporal convolutional network (ITCN) method, is employed. Finally, the enhanced sand cat swarm optimization (ISCO) method-based hyperparameter selection procedure is executed to optimize the detection outcomes of the ensemble models. The experimentation of the OEMPTER-ISCSO technique is accomplished using emotion detection from a text dataset. The key contribution of the OEMPTER-ISCSO technique is listed below.

- The OEMPTER-ISCSO model undergoes a pre-processing process that includes cleaning, normalization, and tokenization to enhance data quality. This process confirms the removal of noise and irrelevant data, making the input more consistent. It significantly improves the efficiency of emotion detection by enhancing the quality of extracted features.
- The OEMPTER-ISCSO method utilizes FastText-based word embeddings to capture the semantic and contextual variances of words, allowing for richer textual representation. This approach facilitates the encoding of sub-word data, thereby enhancing comprehension of rare and misspelt words. The model plays a crucial role in improving the performance of downstream emotion classification models.
- The OEMPTER-ISCSO approach implements an ensemble framework by integrating EDBN, ELNN, and ITCN models. This hybrid setting utilizes every individual model’s merit to capture spatial and temporal features effectively. As a result, it significantly improves the accuracy and robustness of emotion classification.
- The OEMPTER-ISCSO methodology utilizes the ISCO approach to fine-tune the hyperparameters of the ensemble architecture. The ISCO model improves the convergence rate and exploration capabilities by simulating adaptive search behaviours. This results in enhanced model accuracy, mitigated training time, and improved overall efficiency.
- The OEMPTER-ISCSO model is novel in integrating a hybrid ensemble architecture incorporating EDBN, ELNN, and ITCN methods with the ISCO model for hyperparameter tuning. This fusion, combined with FastText-based word embeddings, offers a novel approach to textual emotion detection. Such a configuration has not been previously explored in this domain, resulting in improved accuracy and robustness.

## Related works

Islam et al.^11^ proposed a model using advanced sensor applications and ML techniques. Utilized techniques include ontology-based knowledge representation, multimodal sensor fusion, and DL approaches such as convolutional neural networks (CNN) and recurrent neural networks (RNN). Romero and Armenta^12^ introduced a technique utilizing real-time image processing. The approach utilizes a CNN for facial feature extraction and emotion classification, deployed on a Raspberry Pi 3b+ platform to ensure low-cost and efficient real-time implementation. Asha et al.^13^ developed a modern voice-controlled assistant incorporating voice recognition as well as natural language processing (NLP) for effective identification and essentially retrieving main words, as well as DL models for effective analysis. It takes user voice commands, performs web searches, opens Google Chrome, and converts text to speech. Computer vision (CV) capabilities include real-world object detection and web scraping for pricing data. Emotion detection utilizing pre-trained methods improves human-centric interaction. Pavithra et al.^14^ introduced the SER method enlargement by employing ML models, especially RNN and DL. By examining critical audio features such as prosody, pitch, and rhythm, this method aims to achieve precise emotion recognition for innovative speech instances. Brilli et al.^15^ developed AIris, an AI-powered wearable device that offers VIPs environmental awareness and interaction capabilities. AIris associates an advanced camera fixed on eyewear with an NLP interface, allowing users to receive real-world auditory explanations of their settings. This work also made a functional prototype method that works effectually in real-world circumstances. Bertacchini et al.^16^ proposed a model that integrates the Pepper robot with the chat generative pre-trained transformer (ChatGPT) for real-time, natural language dialogue. Utilizing human-robot interaction (HRI) and NLP, the work simulates interactions with individuals diagnosed with autism spectrum disorder (ASD). Reddy et al.^17^ presented an innovative method that associates hand gesture recognition (HGR) with real-world voice output, developed to help people with paralyzed hands monitor and enhance their hand movements. This novel method utilizes progressive technologies to bridge the distance between action and intention for individuals with inadequate hand mobility. This work represents a significant advancement in assistive technology. Begum et al.^18^ proposed a sign language translation system that utilizes a quantized You Only Look Once version 4 tiny (YOLOv4-Tiny) model for detecting 49 Bengali sign characters, and an LSTM network for generating meaningful text from recognized characters. Kandula et al.^19^ presented a sign language recognition (SLR) system that utilizes webcam-recorded hand gestures to enhance communication for individuals with hearing or speech impairments. Di Luzio, Rosato, and Panella^20^ proposed a method to strengthen emotion classification through video analysis by utilizing explainability models to optimize facial landmark features. Deep models, such as 2D-CNN and deep neural networks (DNNs), are employed, along with an improved integrated gradient method, to detect and refine crucial facial points. This approach enhances accuracy while minimizing noise and reducing computational costs.

Slade et al.^21^ proposed a SER model by integrating the audio spectrogram transformer (AST) with DL techniques such as 1D CNN (1D CNN), Bidirectional LSTM (BiLSTM), and CNN BiLSTM, optimized using a novel cluster search optimization (CSO) technique. CSO utilizes cluster centroid search, reinforcement learning (RL), and noise tempered K-means (NTKM) to enhance model performance across multiple emotion datasets. Neeraja et al.^22^ developed an effective driver somnolence detection system by utilizing DL methods integrated with CV and physiological signal analysis. ML models are integrated to improve detection precision and scalability. Ali and Hughes^23^ proposed an efficient emotion recognition model utilizing a unified biosensor–vision multimodal transformer (UBVMT) model, which integrates self-supervised learning techniques, including masked autoencoding and contrastive modelling. By incorporating 2D representations of ECG/PPG signals with facial features, the model mitigates memory load through homogeneous Transformer blocks, enabling scalable emotion classification in the arousal-valence space. Paul et al.^24^ proposed a real-time attendance system that integrates facial recognition and emotion detection using a dual-path architecture. It leverages ResNet-50 for face recognition, the Vision Transformer (ViT) for emotion detection, and a custom dataset. Choi, Zhang, and Watkins^25^ presented a novel variant of the self-supervised audio spectrogram transformer (SSAST) model. The approach integrates dual representations from both middle and final layers using mean, max, and min patch-wise pooling, improving feature richness and accuracy across multiple benchmark datasets. Wang and Chai^26^ enhanced personalized learning path optimization and learning efficiency by proposing the LSTM-Transformer model. This model utilizes LSTM to capture learners’ behavioural sequences and the Transformer’s self-attention mechanism to enhance context understanding, enabling accurate prediction and adaptive optimization of individual learning trajectories. Ramani et al.^27^ explored emotion detection using a deep Bidirectional LSTM on multimodal mobile sensor data, eliminating the need for manual feature engineering and demonstrating its efficiency for human-robot interaction applications. Prithi and Tamizharasi^28^ improved customer relationship management (CRM) by integrating facial expression recognition into the customer information system (CIS) using a feature fusion deep multi-layer classification (FFDMLC) model. The model employs DL methods for feature computation and classification, with hyperparameters optimized using the COOT optimization algorithm to enhance recognition accuracy. Selvaraju et al.^29^ presented a real-time system for Indian SL and speech-to-text translation in video conferencing using CNN, YOLOv5, Hidden Markov Model (HMM), and WebRTC, improving communication accessibility for the deaf and speech-impaired. Ghadami, Taheri, and Meghdari^30^ utilized transformer encoder-based networks with early and late fusion techniques, optimized by a genetic algorithm (GA), to recognize Iranian Sign Language words. Key features such as hand and lip keypoints, along with spatial metrics, are used for training the model using multi-task learning, enabling accurate word and sentence recognition.

Khanum et al.^31^ proposed an IoT-based wearable device for women’s safety, enabling real-time audio tracking, location monitoring, and emergency alerts, even in offline conditions. Siju and Selvam^32^ developed an HGR system by employing Google Mediapipe to extract 21-point hand landmark vectors, which are later utilized for training a lightweight DNN in TensorFlow. The model recognizes various gestures and is examined in real-time with a live webcam stream, making it appropriate for edge devices. Naik et al.^33^ developed a robust and reliable multimodal emotion recognition system by utilizing DL models across text, audio, and video data. The model integrates Bidirectional Encoder Representations from Transformers (BERT) for text-based emotion detection. The Term Frequency-Inverse Document Frequency (TF-IDF) technique is also utilized for feature extraction. Furthermore, the CNN with audio augmentation for audio signals and the CNN with OpenCV are used for real-time facial expression analysis in video. Liu et al.^34^ introduced a model that employs an Adaptive Evolutionary Computational Integrated Learning Model (AdaECELM), integrating TF-IDF for feature selection, Cuckoo Search Optimisation (CSO), and AdaBoost for ensemble learning through soft voting. Filahi et al.^35^ presented a technique by using diverse ML methods comprising logistic regression (LR), naïve bayes (NB), support vector machine (SVM), random forest (RF), and adaboosting, and DL models like gated recurrent unit (GRU) and long short-term memory (LSTM). Sandulescu et al.^36^ developed NeuroPredict, an AI-driven healthcare platform that utilizes Internet of Medical Things (IoMT) devices and AI models. The technique also integrates AI-based predictive models with voice-based emotion detection algorithms, employing voice features as non-invasive indicators of mental health changes. Muhammad et al.^37^ introduced the CNN technique by integrating transformer models, such as DeBERTa-v3-large, Electra, XLNet, RoBERTa, and T5, to improve model performance in recognizing complex emotional variations. Also, the International Survey on Emotion Antecedents and Reactions (ISEAR) dataset was utilized for testing the model. Thiab, Alawneh, and Mohammad^38^ proposed a method by utilizing DL and transformer-based models. RNNs and transformer architectures are evaluated individually, and their outputs are integrated by using an ensemble learning approach with majority voting to improve performance. Kumar, Khan, and Choi^39^ developed a novel methodology by employing a hybrid DL technique integrating RoBERTa with parameter-efficient adapter layers, Bidirectional LSTM (BiLSTM), and attention mechanisms (AM). Geethanjali and Valarmathi^40^ proposed a hybrid model, the Improved Chimp Optimisation Algorithm–CNN-LSTM (IChOA-CNN-LSTM). The technique is examined under the GeoCoV19 dataset. Arbaizar et al.^41^ utilized Hidden Markov Models (HMM) for handling missing data and a transformer DNN for multivariate time-series forecasting, incorporating classification algorithms to predict emotional valence and responses to psychiatric questionnaires. Kohneh Shahri, Afshar Kazemi, and Pourebrahimi^42^ presented a technique by using AI methods, including DL-based motion detection, body language recognition, image processing, sound and text processing, CV, and NLP. Table 1 summarises the existing studies on emotion recognition for individuals with disabilities.

**Table 1:** Summary of existing studies comprising methods, datasets, and key findings.

**Reference Number**	**Objective**	**Method**	**Dataset**	**Measures**
Islam et al.^11^	To explore how ML and sensor technologies enhance emotion perception and activity recognition.	Multimodal Sensor Data, DL, Temporal–Spatial Behaviour Modelling, Cognitive and Affective Computing	AAL or Smart Home Sensor Dataset	Quality of Life, Emotion Recognition Accuracy, Activity Detection
Romero and Armenta^12^	To develop a real-time model for detecting and classifying seven facial emotions in children.	Camera input, CNN on Raspberry Pi 3b+, Trained CNN model	FER‑2013	Facial Emotion Identification Rate
Asha et al.^13^	To develop a modular, voice-controlled AI assistant for seamless human–machine interaction.	NLP, Voice Commands	Pre-Trained Models	Real-Time Performance, Interaction Accuracy
Pavithra et al.^14^	To develop a DL-based SER system for accurate and reliable emotion detection from speech.	Audio Feature Extraction, DL, RNN	Labelled Emotional Speech Samples	Accuracy, Reliability
Brilli et al.^15^	To develop AIris, an AI-powered wearable device for visually impaired users.	Object Recognition, Scene Interpretation, NLP, Real-Time Auditory Feedback	Real-World Visual Data	Accuracy, Usability
Bertacchini et al.^16^	To explore the use of a Pepper robot integrated with ChatGPT.	Pepper–ChatGPT Integration, Simulated Interaction Scenarios, Social Robotics Dialogue	Simulated ASD Interaction Scenarios	Feasibility, Acceptability, Effectiveness
Reddy et al.^17^	To develop an assistive system to support individuals with paralyzed hands.	HGR, Real-time Voice Output, Sensor-based Input Processing	Custom Gesture Dataset	Accuracy, Responsiveness
Begum et al.^18^	To develop an end-to-end system to aid communication for hearing-impaired individuals.	Quantized YOLOv4-Tiny Detection, Character to Text Generation, LSTM-based Text Model	BdSL 49 Dataset	mAP, Accuracy
Kandula et al.^19^	To bridge communication gaps and memorize complex sign systems.	Webcam hand gesture capture, Gesture recording pipeline, Model training and testing	Custom Webcam Gesture Data	Accuracy
Di Luzio, Rosato, and Panella^20^	To enhance emotion classification via video by optimizing facial landmark inputs.	Facial Landmarks Detection, Binary DNN, Improved Integrated Gradient	Facial Video Dataset	Higher Accuracy, Reduced Cost
Slade et al.^21^	To enhance SER accuracy and robustness.	AST with CSO, Optimizable 1D‑CNN, BiLSTM, CNN‑BiLSTM with Attention, NTKM	EMO‑DB, SAVEE, TESS	Accuracy, mAP
Neeraja et al.^22^	To develop an accurate and real-time system for detecting driver somnolence, thereby improving road safety.	CV, Physiological Signal Monitoring, ML	Driver Drowsiness Datasets	Accuracy, Real-Time Performance
Ali and Hughes^23^	To develop a model for emotion recognition using self-supervised pretraining techniques.	UBVMT, Self-supervised pretraining, masked autoencoding, contrastive modelling, transformer architecture	CMU-MOSEI, Public Biosensor Datasets	Accuracy, Memory Efficiency
Paul et al.^24^	To develop a real-time attendance system for enhanced accuracy and practical deployment.	ResNet-50 Face Recognition, ViT Emotion Detection, Dual-Path Architecture, Web Integration	Custom Real-Time Dataset	Accuracy, AUC-ROC Score
Choi, Zhang, and Watkins^25^	To enhance audio classification for improved performance.	SSAST, Multi-Layer Feature Fusion, Patch-Wise Pooling, Self-Supervised Learning, Dual Representation	CREMA-D, TESS, RAVDESS, Speech Emotion Classification, Isolated Urban Events, CornellBirdCall	Accuracy Rates
Wang and Chai^26^	To optimize personalized learning paths and learning efficiency.	LSTM Behaviour Capture, Transformer Self-Attention, DL Integration	Learner Behaviour Sequences	Knowledge Mastery, Learning Time, Satisfaction
Ramani et al.^27^	To accurately detect human emotions without manual feature engineering.	Deep Bidirectional LSTM, Multimodal Sensor Fusion, Iterative DL	On-Body, Ambient, Geographical Sensors	Accuracy, Effectiveness
Prithi and Tamizharasi^28^	To enhance CRM for accurate emotion analysis.	FFDMLC, Feature Fusion, DL, COOT	CK+, FER2013	Recognition Rate, Accuracy
Selvaraju et al.^29^	To develop a real-time ISL gesture-to-text subtitle system for individuals with hearing and speaking impairments.	CNN, YOLOv5, HMM, WebRTC	ISL Gesture Video Dataset	Accuracy, Latency, Usability
Ghadami, Taheri, and Meghdari^30^	To develop a transformer-based DL system for improved communication and learning.	Early And Late Fusion Transformers, GS, Keypoint Feature Extraction, Multi-Task Learning	101 Iranian Sign Language Words	Accuracy, Real-Time Feedback
Khanum et al.^31^	To develop an IoT-based wearable device to enhance women’s safety, including offline functionality for evidence preservation.	IoT Wearable Device, Real-Time Audio Tracking, Location Tracking, Emergency Alert System	Not Specified/Real-Time User Data	Response Time, Alert Accuracy
Siju and Selvam^32^	To develop a system for accurate and efficient SLR compatible with edge devices.	Google Mediapipe Landmarks, DNN, Tensorflow Training, Live Webcam Testing	Hand Gesture Images (Peace, Okay, Stop)	Accuracy, Latency
Naik et al.^33^	To develop a robust multimodal real-time emotion recognition system.	Text: BERT + TF-IDF, Audio: CNN + Augmentation, Video: CNN + OpenCV	Four Kaggle Datasets (audio, video, text)	Audio and Video Accuracy of 99.44% and 97.66%, Audio and Video Validation of 94.71% and 65.38%
Liu et al.^34^	To enhance sentiment analysis accuracy for short texts.	TF-IDF, CSO, SVM, AdaBoost Soft Voting	Six Real Polar Sentiment Analysis Datasets	Accuracy Improvement: >4.5%
Filahi et al.^35^	To improve e-commerce decision-making using IoT data and ML models.	LR, NB, SVM, RF, AdaBoosting, GRU, LSTM	Customer Behaviour and Preference Data Collected Via IoT Devices	Accuracy of 88%, F1-Score of 0.927, Precision of 0.908, Recall-0 of 0.569 and Recall-1 of 0.947
Sandulescu et al.^36^	To develop an AI-driven healthcare platform.	IoMT Sensor Integration, AI Predictive Models, Emotion Detection Algorithm	Patient Sensor Data and Voice Recordings	Early Symptom Detection, Disease Progression Tracking
Muhammad et al.^37^	To enhance emotion detection accuracy on imbalanced and limited data.	DeBERTa-v3-large + CNN, Electra + CNN, XLNet-base, RoBERTa + CNN, T5-base, Synonym Replacement Augmentation	ISEAR	Accuracy (best) of 94.94%, Accuracy (others) of 93%/69%, Improved Precision & Recall
Thiab, Alawneh, and Mohammad^38^	To evaluate and enhance emotion classification in textual conversations.	RNN and Transformer-based Models, Ensemble via Majority Voting	SemEval 2019 Task 3 (EmotionX)	Transformer F1-Score of 75.55%, RNN F1-Score of 67.03%, Ensemble F1-Score of 77.07%
Kumar, Khan, and Choi^39^	To develop an accurate mental health detection model from social media text.	RoBERTa + Adapter Layers, BiLSTM, AM	Filtered GoEmotions Dataset	Accuracy (binary): 92%, Accuracy (multiclass): 88%
Geethanjali and Valarmathi^40^	To improve multimodal sentiment analysis during the COVID-19 pandemic.	CNN, LSTM, IChOA, Feature Fusion Strategy	GeoCoV19 Dataset	Accuracy of 97.8%
Arbaizar et al.^41^	To enable real-time, objective monitoring and prediction of psychiatric patients’ emotional states.	HMM, Transformer DNN, Time-Series Forecasting, Classification Algorithms	Passive and Self-Reported Data from the Evidence-Based Behaviour (eB2) App	Emotional state accuracy: 0.93, ROC AUC (valence): 0.98, ROC AUC (1-day prediction): 0.87, Accuracy (suicidal ideation): 0.9, ROC AUC (suicidal ideation): 0.77
Kohneh Shahri, Afshar Kazemi, and Pourebrahimi^42^	To develop and evaluate a comprehensive sentiment analysis model for improved accuracy and speed.	Image, Sound and text processing, CV, NLP	Social Network Multimedia Data	Accuracy (High), Speed (Fast)

Despite crucial improvements in DL, CV, and transformer-based models across various emotion recognition, SL, and safety applications, several limitations still exist. Many models require large annotated datasets, which are often scarce, resulting in challenges to generalization and robustness. Few models exhibit inefficiency due to high computational complexity and memory requirements on resource-constrained edge devices. Existing multimodal fusion approaches often encounter issues with synchronization and effective feature integration, which can impact accuracy. Furthermore, few systems comprehensively address offline functionality and privacy concerns, particularly in safety-critical applications. There is also limited research on adaptive models that can dynamically optimize performance based on varying input quality and user contexts. Despite these improvements, various techniques still encounter threats due to their reliance on specific datasets, restricted generalizability across diverse data sources, and challenges in handling noisy or imbalanced data. Moreover, a few models increase computational complexity. The research gap in addressing these concerns involves developing lightweight, scalable architectures capable of efficient multimodal fusion, enhancing both offline and real-time capabilities, and improving model adaptability with minimal manual intervention. Moreover, many models rely heavily on manual feature engineering, which limits scalability and adaptability across diverse datasets and applications. Addressing this research gap requires the design of end-to-end self-supervised or semi-supervised learning frameworks that reduce dependency on labelled data while maintaining high accuracy and efficiency. Additionally, a gap exists in developing scalable and efficient systems that support high accuracy across diverse real-world scenarios while effectively managing data heterogeneity and model complexity.

## Proposed methodology

**Fig. 1:**
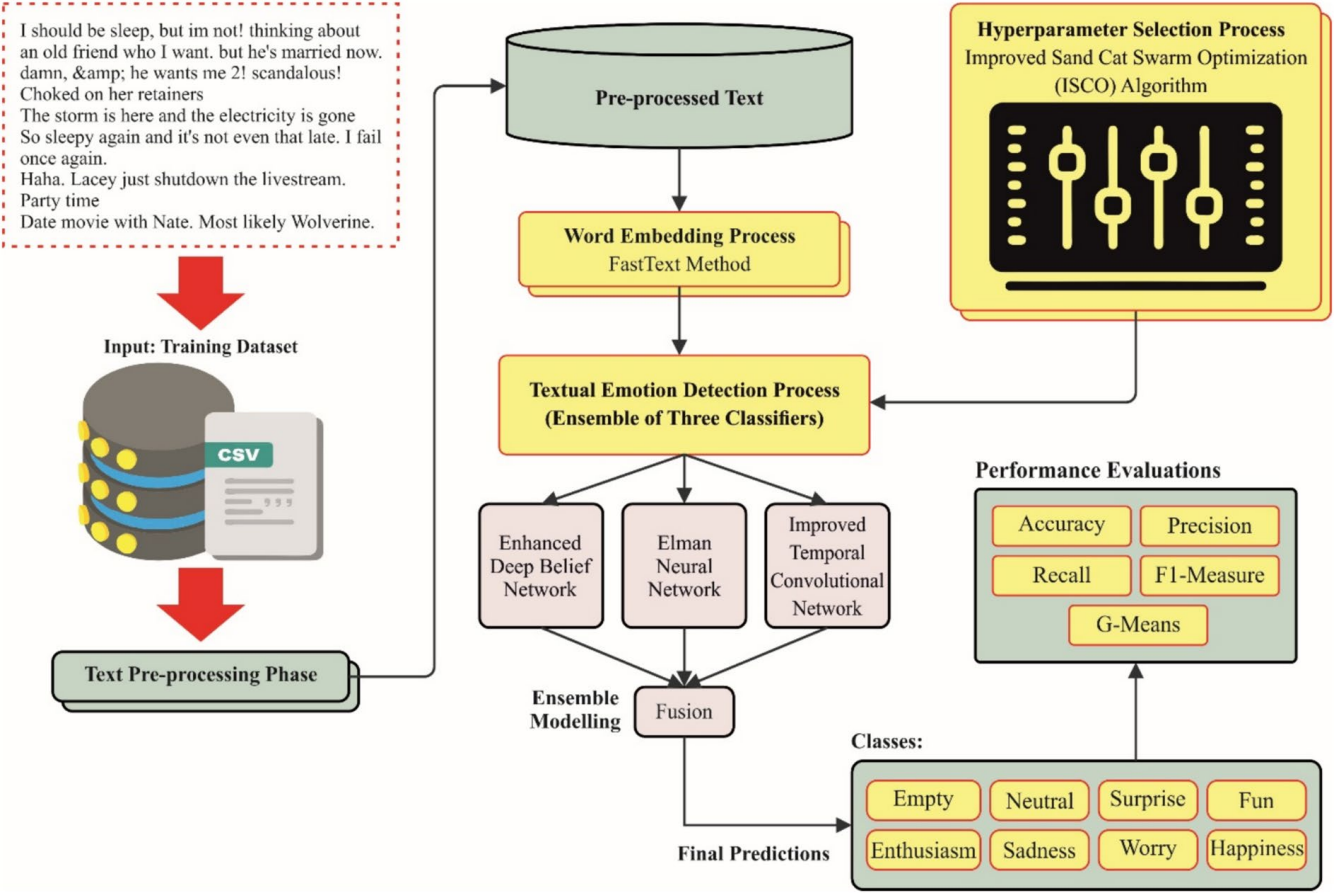
Overall working process of OEMPTER-ISCSO model.

This study presents an OEMPTER-ISCSO model. The primary objective of the OEMPTER-ISCSO method is to enhance the communication of individuals with disabilities by accurately recognizing emotions in text. The proposed OEMPTER-ISCSO method comprises several stages, including text pre-processing, word embedding, classification, and hyperparameter tuning. The overall working flow procedure of the OEMPTER-ISCSO method is portrayed in Fig. 1.

### Text pre-processing

Initially, the text pre-processing stage involves multiple levels to normalize and clean the input text^43^. Text pre-processing transforms text into a design that is analyzable and predictable for specific tasks. This includes eliminating unimportant data, such as stop-words, URLs, stemming, and lemmatization, and executing tokenization, which removes unrelated data and prepares the dataset for further processing. The pre-processing stages in text summarisation are determined according to the task’s goal, such as removing related features and eliminating unrelated data to enhance the algorithm’s performance. It is crucial to achieve optimal outcomes in text summarisation, as they directly impact the accuracy and quality of the produced analyses. The pre-processing phase plays a vital role in converting raw text into a structured format appropriate for feature extraction and classification. Stemming mitigates words to their root forms by removing suffixes, often resulting in non-lexical stems. In contrast, lemmatization considers the context and reduces words to their dictionary base form, thereby improving the quality of extracted features. By applying these targeted pre-processing techniques, the system effectually captures semantic, statistical, and linguistic characteristics, thereby enhancing the accuracy and coherence of emotion summarisation.

The initial data cleaning phase involves removing duplicate URLs, handling dynamic URLs, and preserving those with embedded HTML components to ensure that only English-language-related content is retained for evaluation. Removing stop words—common words like "a," "an," and “the”—helps mitigate noise and improves concentration on crucial terms that carry semantic weight. The tokenization procedure further simplifies the text by separating large blocks into small units, such as splitting sentences into individual words for easier analysis.

The stemming procedure is applied to mitigate words to their root form, which may not be an actual word but captures the base idea of related terms. For example, "running," "runner," and “ran” are reduced to “run”. This process helps integrate semantically identical words and enhances text analysis effectively. Lemmatization, on the contrary, refines this process by altering words to their proper base form using a dictionary, ensuring grammatical accuracy and preserving the original context. For instance, from "sharing," lemmatization provides the correct base form "share," contributing to more precise and meaningful text summarisation. This process mitigates redundancy and enhances consistency in the analysis, allowing for a more precise and accurate representation of emotions.

### FastText-based word embedding

**Fig. 2:**
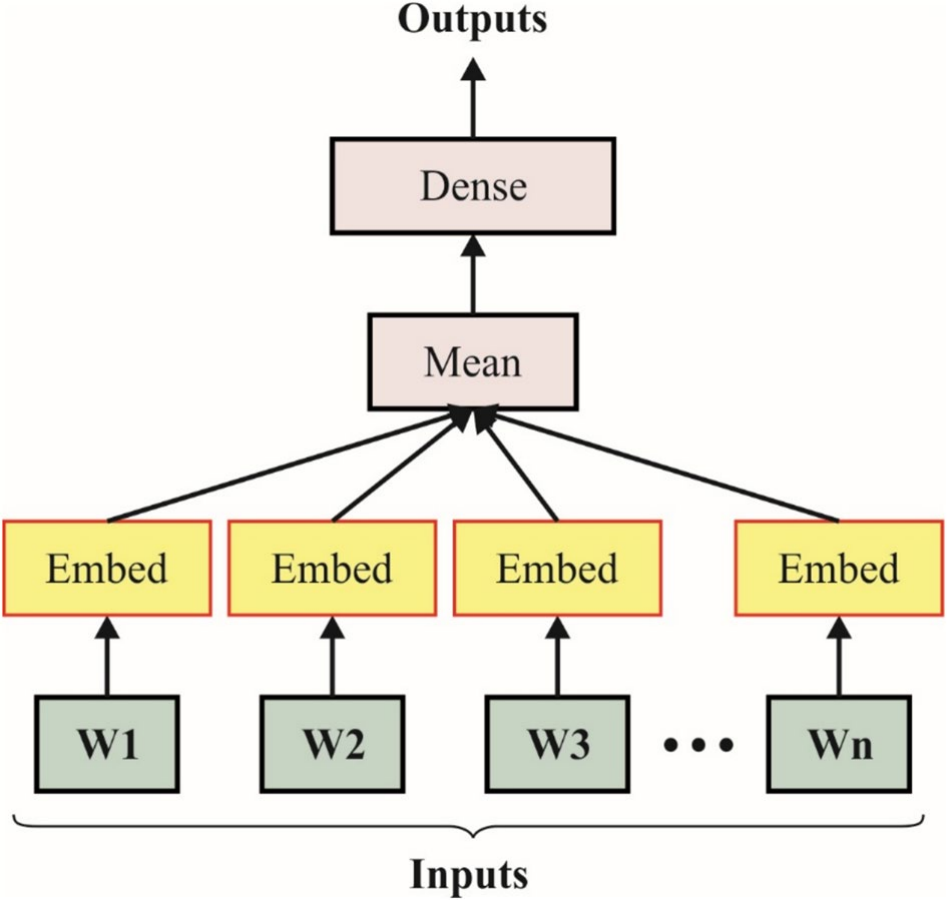
Architecture of FastText model.

Next, the FastText method is employed for the word embedding process, transforming words into numerical vector representations^44^. This model is chosen due to its capability to capture subword data, which is advantageous for handling out-of-vocabulary (OOV) words and morphologically rich languages. This technique depicts words as a sum of character n-grams, facilitating the comprehension of the internal word structures, unlike conventional embeddings such as Word2Vec or GloVe. This improves its robustness in noisy or domain-specific datasets. Furthermore, the model is computationally effective and gives meaningful vectors even for rare or misspelt words. Its pre-trained models on large corpora contribute to enhanced semantic and contextual representation, making it an ideal choice for downstream NLP tasks, such as emotion detection. Fig. 2 illustrates the flow of the FastText model.

It is a word representation in a vector space that captures semantic relations among words. Specifically, it is a mathematical method that characterizes words such that related words are positioned closer together in the vector space. These representations are frequently applied in ML and NLP tasks. The notion after word embeddings is to transform words into mathematical vectors to safeguard their semantical relationships. This enables ML models to understand meanings and healthier relationships more effectively. Word embeddings mainly benefit sentiment analysis, text classification, and language translation tasks.

FastText is a lightweight, free, and open-source library designed for effective text representation and classification. It is designed to process large text databases efficiently and is primarily suitable for tasks such as word embedding, language identification, and text classification. FastText can constantly represent vectors (embeddings) for words in all texts. These embeddings utilize semantic data and are beneficial for various NLP tasks. It assists in training the text classifier using a shallow neural network (NN). This makes it efficient for tasks when labelled data is presented for training, such as topic classification or sentiment analysis.

The fastText model is derived from an NN structure that combines the Bag-of-Words (BoW) model and sub-word data. The formulations are stated as exposed in Eq. (1).

1 $$-\frac{1}{N}{\sum }_{n=1}^{N}{y}_{n} log\left(f\left(BA{x}_{n}\right)\right)$$

Where are the standardized features bag of the $nth$ document, the labels $A$ and $B$, and the weighted matrices? This approach is trained asynchronously on numerous CPUs with a linearly decaying learning rate and stochastic gradient descent. The training process involves updating the NN parameters to minimize these objective functions. It utilizes negative sampling and hierarchical softmax models to make the training process more effective. It is essential to understand that, although these provide an overall review, the actual performance details, optimizations, and hyperparameters may differ according to the specific settings and version used in FastText.

### Classification using ensemble models

An ensemble of three classifiers is employed for textual emotion detection and classification: the EDBN model, ELNN technique, and ITCN method. The ensemble model is chosen to utilize the unique merits of every model, improving overall performance in textual emotion detection. The model is prevalent because it can effectively capture hierarchical and abstract feature representations, enhancing emotion recognition from complex text patterns. ELNN, with its feedback connections, outperforms modelling temporal dependencies in sequential data. ITCN offers the benefits of capturing long-range dependencies with reduced complexity and faster training compared to RNNs. Altogether, these models complement each other, confirming enhanced generalization, robustness, and classification accuracy over single-model approaches, particularly in emotionally diverse and context-sensitive textual datasets.

#### EDBN classifier

Comparable to the conventional DBN, the EDBN learning process primarily consists of two phases: tuning and pretraining. During this pretraining phase, the contrastive divergence (CD) model is applied to execute unsupervised training for the RBM^45^. Previously, the complete NN was extended into a forward-form network during the fine-tuning phase, and the weights of the complete network were adjusted by accepting the EBP model. The probability equation for the activation state of the basic component RBM is exposed as Eqs. (2-3):

2 $$\begin{array}{c}p({h}_{j}=1|v)=\sigma \left({\sum }_{j=1}^{m}{w}_{ji}{v}_{j}+{b}_{j}\right)\\ =\frac{1}{1+{e}^{-\left({\sum }_{j=1}^{m}{w}_{ji}{v}_{j}-{b}_{j}\right)}}\end{array}$$

3 $$\begin{array}{c}p({v}_{j}=1|h)=\sigma \left({\sum }_{j=1}^{m}{w}_{ji}{h}_{i}+{a}_{j}\right)\\ =\frac{1}{1+{e}^{-\left({\sum }_{j=1}^{n}{w}_{ji}{h}_{i}-{a}_{j}\right)}}\end{array}$$

Whereas ${v}_{j}$ designates the input of the $jth$ node within the visual layer, ${h}_{i}$ characterizes the $ith$ node value of the hidden layer (HL), ${a}_{j}$ and ${b}_{i}$ represent offset values of the hidden and visible neurons in sequence, ${w}_{ji}$ refers to the connection weight between the hidden neuron $i$ and the visible neuron $j$, $sigmoid$ signifies the activation function, and the sigmoid expression is $1/(1+{e}^{-x}),$ $m$ stands for visible neuron counts, $n$ indicates the hidden neuron counts. Let parameters $\theta =(w, a,b)$, the update rule of weight and offset is shown as

4 $${\theta }^{(p+1)}={\theta }^{(p)}+\Delta \theta =\langle {h}_{i}^{0}{v}_{j}^{0}\rangle -\langle {h}_{i}^{1}{v}_{j}^{1}\rangle$$

Whereas $\langle \cdot \rangle$ characterizes the average value gained from the sampling state, ${h}_{i}^{0}{v}_{j}^{0}$ signifies the primary state distribution, ${h}_{i}^{1}{v}_{\dot{j}}^{1}$ specifies the state gained after a Markov iteration, and $p$ signifies the sum of unsupervised training.

During this EDBN, ${p}_{i}$ and ${p}_{j}$ correspond to the $ith$ hidden neuron and the $jth$ visible layer node, respectively. The sigmoid activation function in Eqs. (2-3) are reserved and eliminated. The constant transformation of Eqs. (2-3) are understood by including a zero‐mean Gaussian noise to the input of the sigmoid activation function of the samples; the expressions after the transformation are exposed in Eqs. (5-6):

5 $${p}_{i}={\phi }_{i}\left({\sum }_{j}^{m}{w}_{ji}{p}_{j}+\beta \cdot {N}_{i}\left(\text{0,1}\right)\right)$$

6 $${p}_{j}={\phi }_{j}\left({\sum }_{i}^{n}{w}_{ji}{p}_{i}+\beta \cdot {N}_{j}\left(\text{0,1}\right)\right)$$

Whereas,

7 $${\phi }_{i}\left({x}_{i}\right)={\theta }_{l}+\left({\theta }_{h}-{\theta }_{l}\right)\cdot \frac{1}{1+{e}^{-{q}_{i}{x}_{i}}}$$

8 $${\phi }_{j}\left({x}_{j}\right)={\theta }_{l}+\left({\theta }_{h}-{\theta }_{l}\right)\cdot \frac{1}{1+{e}^{-{q}_{{j}^{X}j}}}$$

Eqs. (5) and (6) represent the inference and learning process of the EDBN, $where N(\text{0,1})$ denotes a Gaussian random variable with a mean of 0 and a variance of 1. $\beta$ refers to the constant, $\phi ()$ characterizes the sigmoid function of the asymptotic as ${\theta }_{h}$ and ${\theta }_{l}$, $q$ designates the noise control variable and is applied for controlling the sigmoid function slope. Based on the comparison divergence rule, the updated equations of bias and weight value are presented as shown (9) $-$(11):

9 $$\Delta {w}_{ij}={\alpha }_{w}\left(\langle {p}_{j}^{0}{p}_{i}^{0}\rangle -\langle {p}_{j}^{1}{p}_{i}^{1}\rangle \right)$$

10 $$\Delta a=\frac{{\alpha }_{a}}{{a}^{2}}\left(\langle {p}_{i}^{{0}^{2}}\rangle -\langle {p}_{i}^{{1}^{2}}\rangle \right)$$

11 $$\Delta b=\frac{{\alpha }_{b}}{{b}^{2}}\left(\langle {p}_{j}^{{0}^{2}}\rangle -\langle {p}_{j}^{{1}^{2}}\rangle \right)$$

Now, ${x}_{w},$ ${\alpha }_{a}$, and ${\alpha }_{b}$ characterize the learning rate of NNs.

#### ELNN classifier

**Fig. 3:**
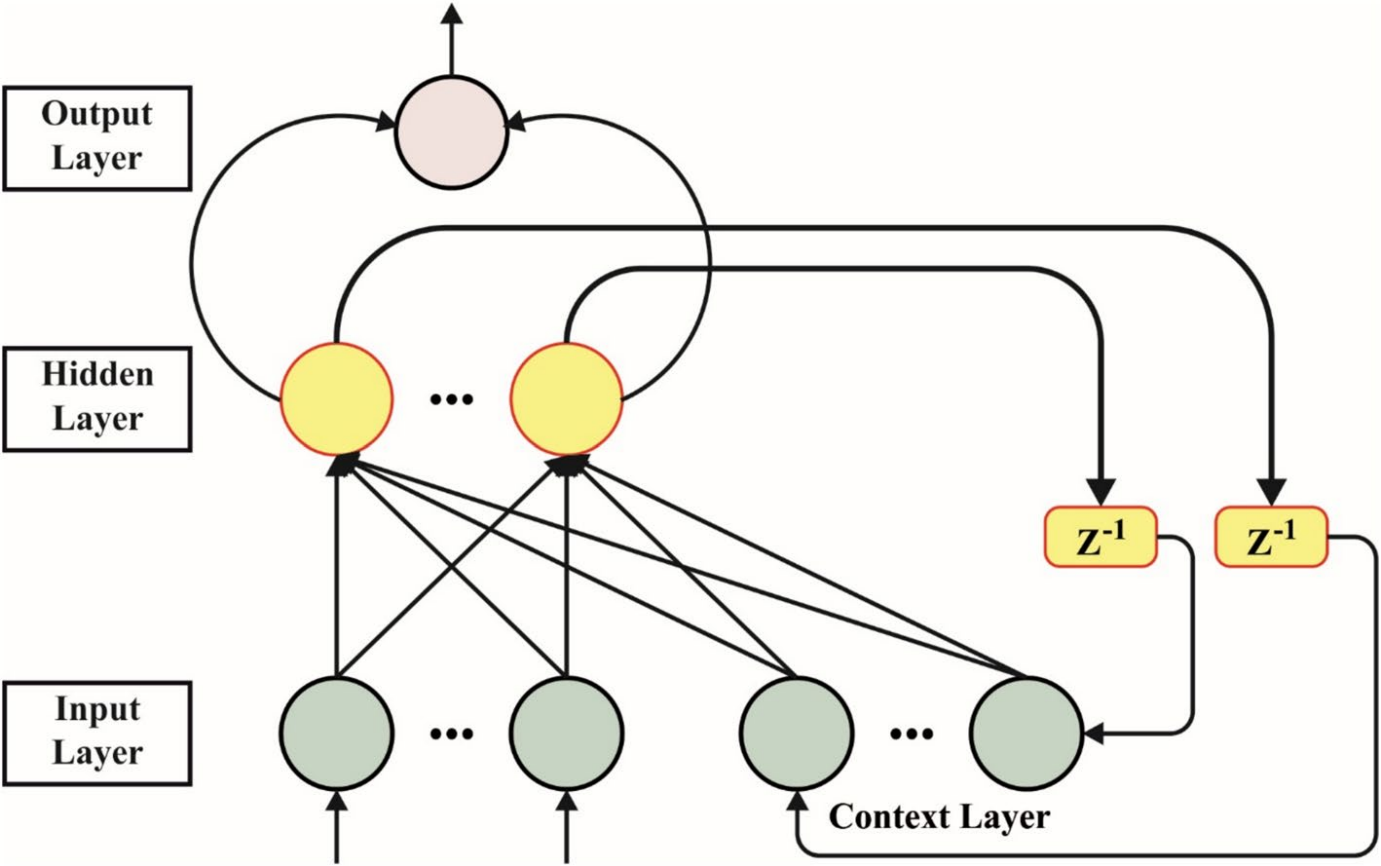
Structure of the ELNN model.

Elman presented the ELNN, NN, as a kind of recurrent NN (RNN) containing many connected neurons^46^. It originated from the significant architecture of the back-propagation NN (BPNN) and includes the added HL. These additional layers function as the one-step delay component, enabling the system to retain a general system configuration recognized according to the associations among neurons. This contains self-organizing, feed-forward, and recurrent NNs. Feedback networks are especially unique in that they transfer data in either direction, either backwards or forward. Response information may affect neurons through various networking layers or be limited to a particular layer. BPNN is an extensively accepted multilayered feed-forward NN through outstanding generalizability and nonlinear feature maps. The training procedure modified the network weights during forward data propagation. The thresholds and weights are adjusted to ensure that the BPNN’s forecast output gradually approaches the target output. The hierarchical structure of the Elman network typically consists of four distinct layers. In the HL, the signals are handled through the activation function. This layer additionally includes feedback characteristics. Ultimately, the output layer determines the outcomes. Fig. 3 portrays the structure of the ELNN model.

#### ITCN classifier

The TCN is utilized in time series prediction by multiple researchers; nevertheless, the difficulty in sparse mode is that it is unable to precisely capture the time series process through the calculation process, resulting in a lower signal-to-noise ratio, and the disappearance or explosion of the gradient may occur during the method’s training^47^. Based on the idea of global average pooling (GAP) and soft thresholding, the ITCN is presented in this research.

Based on the normal residual connections, a sub‐network depends on the threshold inserted into the ITCN. It is presented in Eq. (12):

12 $$f\left(x\right)=\left\{\begin{array}{ll}x+\xi & x<-\xi \\ 0& \left|x\right|\le \xi \\ x-\xi & x>\xi \end{array}\right.$$

In Eq. (12), $\xi$ represents the determined threshold, $x$ signifies input variables, and $f(x)$ denotes the soft thresholding function. Soft thresholding separates the number of input variables that are independent output variables. It significantly defines the $\xi$ value; thus, the sub-network is presented in global mean pooling, which is adaptively determined according to the input variable characteristics.

In the sub-network, the GAP is performed as the dropout layer value of the output. Afterwards, a 1D vector is fed into the fully connected (FC) layers, and the final layer is the $Sigmoid$ function, which normalizes the output values to zero and one. The scaling weight is noted as $\gamma$. The threshold $\xi$ value could be defined as Eq. (12):

13 $$\xi =\gamma GAP\left(\left|x\right|\right)$$

Eq. (13) defines the threshold $\xi$ as the value to which products are set to zero or one. This approach verifies that the threshold is established by the example data features that make the adaptable method and enhance the capability to remove effective features from the input data.

### ISCO-based hyperparameter tuning

**Fig. 4:**
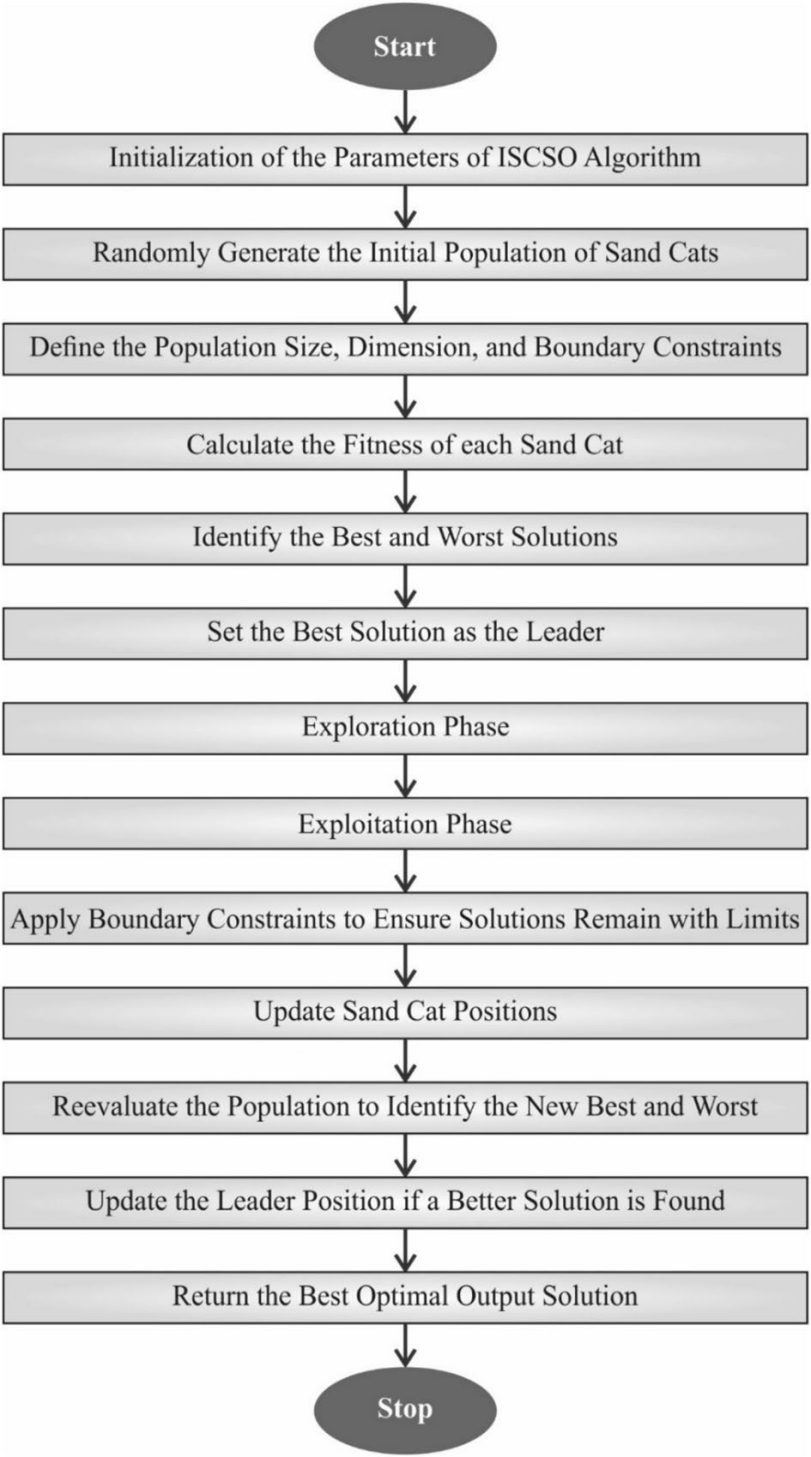
Workflow of the ISCO methodology.

 The ISCO-based hyperparameter selection procedure optimizes the ensemble models’ recognition outcomes^48^. This model is chosen due to its superior exploration-exploitation balance and fast convergence rate. This method dynamically alters search directions using adaptive coefficients inspired by the hunting behaviour of sand cats, which are not typically observed in conventional grid or random search methods. This ensures optimal parameter selection, specifically in high-dimensional search spaces. ISCO also avoids local optima more effectively than standard evolutionary or swarm-based techniques, such as PSO or GA. Its lightweight structure and minimal computational cost make it ideal for fine-tuning complex ensemble models, enhancing accuracy and mitigating overfitting. Fig. 4 illustrates the working flow of the ISCO model.

The SCSO model simulates two key survival behaviours of sand cats: hunting and foraging. Compared to other population-based intelligence models, SCSO exhibits robust optimization abilities appropriate for complex multi-objective problems. However, its search accuracy and convergence speed are limited, making it prone to local optima. To address this, three improvements are utilized for improving its global search capability, resulting in the ISCSO method, which is applied to optimize edge node utilization. Additionally, logistic chaotic mapping is used to initialize populations, leveraging its non-linearity, ergodicity, and randomness to improve convergence speed and precision, as shown in Eqs. (14-15).

14 $${X}_{i+1}=\gamma {x}_{i}+\left(1-{x}_{i}\right)$$

15 $${Y}_{i+1}={l}_{\text{min}}+{X}_{i}\cdot \left({l}_{\text{max}}-{l}_{\text{min}}\right)$$

Here, $\gamma$ helps as the controller parameter, using $\gamma>1$, the range of values ${x}_{i}$ drops inside $0<{x}_{i}<1,$ ${x}_{id}$ indicates the sequence of chaos produced by Eq. (19), ${Y}_{i}$ characterizes the location of $the ith$ individual, and ${l}_{\text{max}}$ and ${l}_{\text{min}}$ symbolize the searching region of the populations. At this stage, the SC identifies prey by evaluating the optimal position, current position, and sensitivity range. This occurrence is accurately explained by Eq. (16):

16 $$pos\left(i+1\right)=r\left(po{s}_{bc}\left(i\right)-rand\left(\text{0,1}\right)\cdot po{s}_{c}\left(i\right)\right)$$

While $po{s}_{bc}(i)$ denotes optimum solutions, $po{s}_{c}(i)$ refers to the present location, and $r$ signifies a sensitivity range. This approach within the model enables the discovery of numerous search routes, helping individuals make effective adjustments to their locations. Next, combined with spiral exploration, individuals implement searching processes inside the searching region in a spiral pattern. Expanding the model’s exploration abilities improves its chances of escaping local optima and enhances its overall global search performance. The updated equation is characterized by Eq. (17):

17 $$pos\left(i+1\right)=0\cdot r\left(po{s}_{bc}\left(i\right)-rand\left(\text{0,1}\right)\cdot po{s}_{c}\left(i\right)\right)$$

18 $$0=exp\left(bg\right)cos\left(2\pi g\right)$$

Eq. (18) indicates the calculation of the features of spiral exploration, signified as $0$. Whereas $b$ signifies the constant of spiral shape and $g$ characterizes the route coefficient, where $g\in [-\text{1,1}].$

During this prey-attacking stage, an arbitrary location, denoted as $p{os}_{ted} (i)$ is generated using the top and current locations. Then, an arbitrary angle $\alpha$ is designated over the roulette model, and the attack process is implemented utilizing Eq. (20):

19 $$po{s}_{rand}\left(i\right)=\left|rand\left(\text{0,1}\right)\cdot po{s}_{bc}\left(i\right)-po{s}_{c}\left(i\right)\right|$$

20 $$pos\left(i+1\right)=po{s}_{bc}\left(i\right)-po{s}_{rand}\left(i\right)\cdot r\cdot cos\left(\alpha \right)$$

The prey attacks in the normal model are performed at arbitrary angles, which may result in the model discounting some optimal solutions. The mathematical formulation is defined in Eq. (21):

21 $$pos\left(i+1\right)=po{s}_{bc}\left(i\right)+\left(po{s}_{bc}\left(i\right)-po{s}_{c}\left(i\right)\right)\cdot C\cdot levy$$

22 $$levy=\frac{u}{|vs{|}^{-\beta }}$$

Here, variables $u$ and $v$ follow normal distributions, $\sim and N\left(0,{\sigma }_{u}^{2}\right),v\sim N\left(0,{\sigma }_{v}^{2}\right)$, and $C$ denote the constant demonstrating the step adjustment coefficient. The comprehensive stages of the ISCSO model are obtainable in Algorithm 1.

#### Algorithm 1:

Pseudocode of the ISCO model
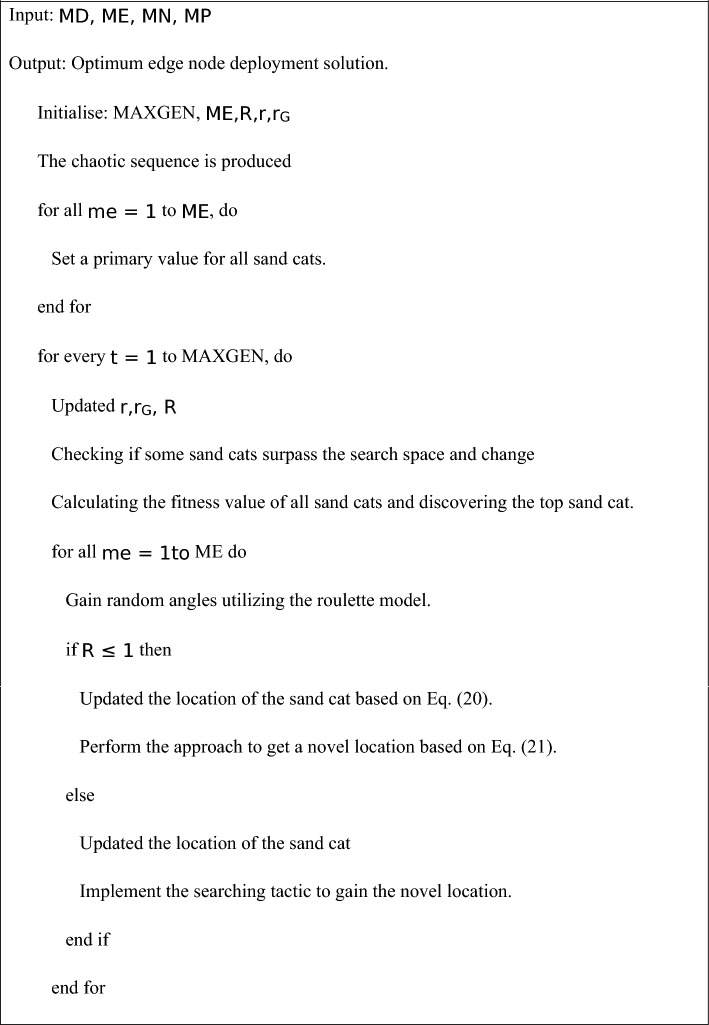


The ISCO model facilitates the effective tuning of the OEMPTER-ISCSO model by utilizing adaptive behaviours inspired by sand cats, including spiral exploration, chaotic initialization, and Levy flights. This modification enhances the global search capability of the model and also prevents local optima, ensuring faster convergence in high-dimensional spaces. ISCO achieves optimal model performance with minimal computational cost by dynamically adjusting paraeters such as learning rate, dropout, and layer configuration. This results in enhanced accuracy, mitigated overfitting, and efficient emotion recognition from text, making the system highly suitable for real-time communication in sustainable environments for individuals with disabilities. Table 2 depicts the hyperparameter values of the OEMPTER-ISCSO technique.

**Table 2:** Key parameters of the ISCO model for tuning the OEMPTER-ISCSO technique in high-dimensional search spaces.

**Parameter**	**Symbol**	**Description**	**Typical Range/Value**
CHAOS_CONT_PARAM	$\gamma$	The range of the chaos sequence is controlled	$\gamma>1$
SEARCH_RANGE_LIMIT	${l}_{\text{max}}$, ${l}_{\text{min}}$	Lower and upper limits of the search space	Problem-Defined
SENSITIVITY_RANGE	$r$	The exploitation-exploration balance is altered	0.1–1
SPIRAL_CONSTANT	$b$	Tightness of the spiral pattern is defined	0.5–2
SPIRAL_ROUTE_CO-EFF	$g$	Spiral direction is determined [−1, 1]	Random Number in [−1, 1]
STEP_ADJUST_CO-EFF	$C$	Step size in the Levy flight is controlled	0.001–1
Levy distribution exponent	$\beta$	The Levy distribution is determined	1.5–2
POPU_SIZE	$N$	Overall, individuals/agents in the populace	20–50
Logistic chaotic map seed	${x}_{0}$	Initial value for chaotic sequence	${0<x}_{0}<1$

Fitness selection is a substantial factor in influencing the outcome of the ISCO model. The hyperparameter range procedure concludes by evaluating the efficiency of the candidate solution encoded in the model. The ISCO model reflects accuracy as a foremost standard for projecting fitness functions. Its formulation is expressed as follows:

23 $$Fitness =\text{ max }(P)$$

24 $$P=\frac{TP}{TP+FP }$$

Here, $TP$ signifies the positive value of true, and $FP$ denotes the positive value of false.

## Experimental analysis

The experimental validation of the OEMPTER-ISCSO approach is examined under the Emotion detection from text dataset^49^. The technique is simulated using the Python 3.6.5 tool on PC i5-8600k, 250GB SSD, GeForce 1050Ti 4GB, 16GB RAM, and 1TB HDD. The parameter settings are: learning rate: 0.01, activation: ReLU, epoch count: 50, dropout: 0.5, and batch size: 5. The dataset consists of 22280 samples below eight sentiments, as shown in Table 3. Table 4 illustrates the sample texts.

**Table 3:** Details of the dataset.

**Sentiment**	**No. of Instances**
Empty	800
Sadness	5000
Enthusiasm	700
Neutral	500
Worry	500
Surprise	2000
Fun	1700
Happiness	5000
**Total Instances**	**22280**

**Table 4:** Sample texts.

**S.no**	**Sentiment**	**Content**
1	Empty	“Have a headache I’m going to bed. Goodnight!”
2	Neutral	“has work this afternoon”
3	Surprise	“my gap year is going so quick”
4	Fun	“Five o’clock can’t come any faster”
5	Happiness	“english class! working on interactive orals”

**Fig. 5:**
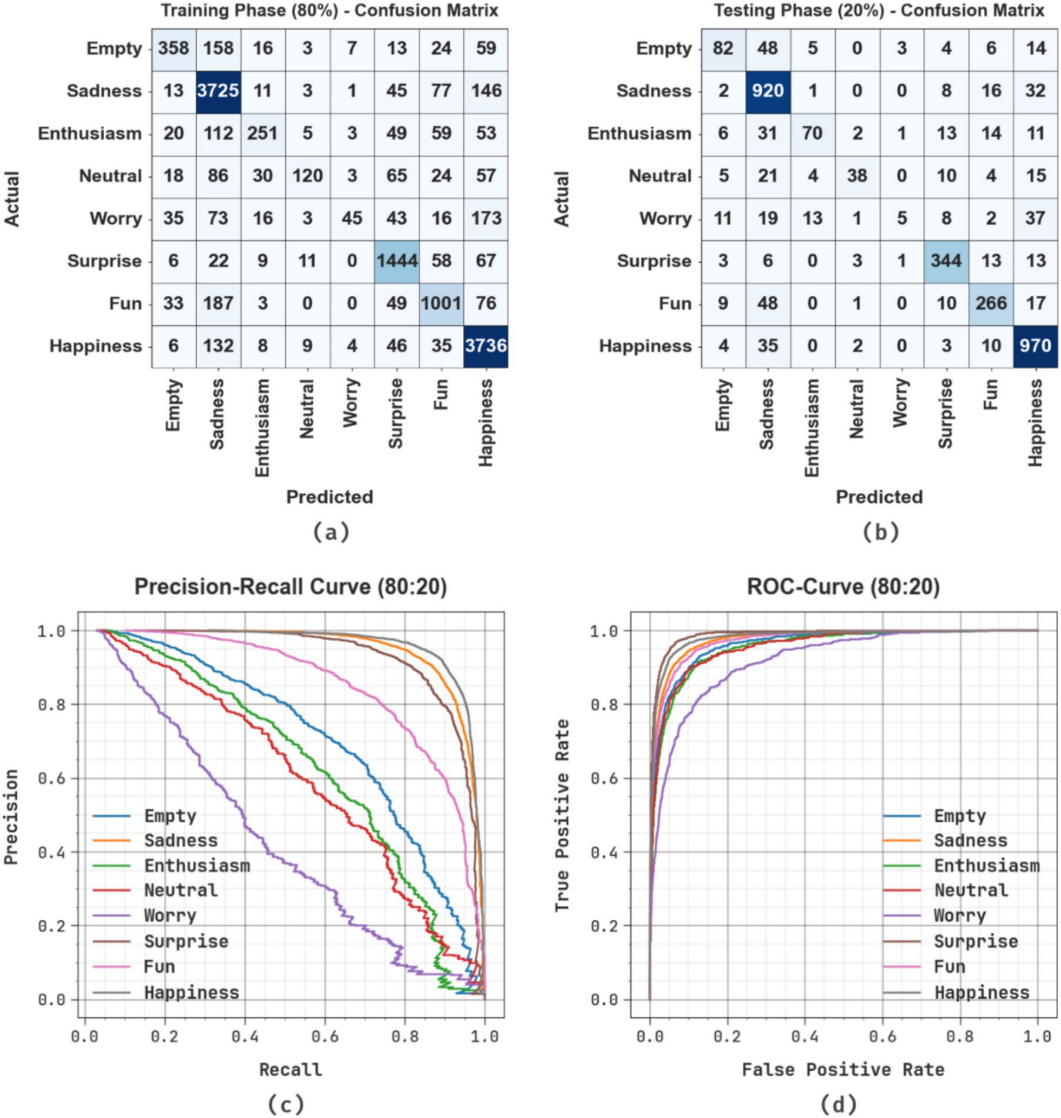
80%TRPH and 20%TSPH of (**a**-**b**) confusion matrices and (**c**-**d**) curves of PR and ROC.

Fig. 5 displays the classifier results of the OEMPTER-ISCSO approach below 80%TRPH and 20%TSPH. Fig. 5a and 5b represent the confusion matrices through precise classification and identification of distinct class labels. Fig. 5c-5d shows the PR and ROC studies, which indicate higher performance across all class labels. The confusion matrix illustrates robust classification for classes such as Sadness, with 3,960 correct predictions, and Happiness, with 3,932 correct predictions. In contrast, classes such as Worry and Neutral have lower TP counts, indicating challenges in these categories. During testing, the model emphasized robust performance with notable TP in Sadness (969) and Happiness (1011), though lower recall is observed for Worry (85) and Neutral (90). The PR and ROC curves exhibit robust TP rates for most classes, illustrating consistently high precision and accuracy. While recall for the Worry and Neutral classes is comparatively lower, the model presents robust and reliable performance across the majority of emotion categories.

**Fig. 6:**
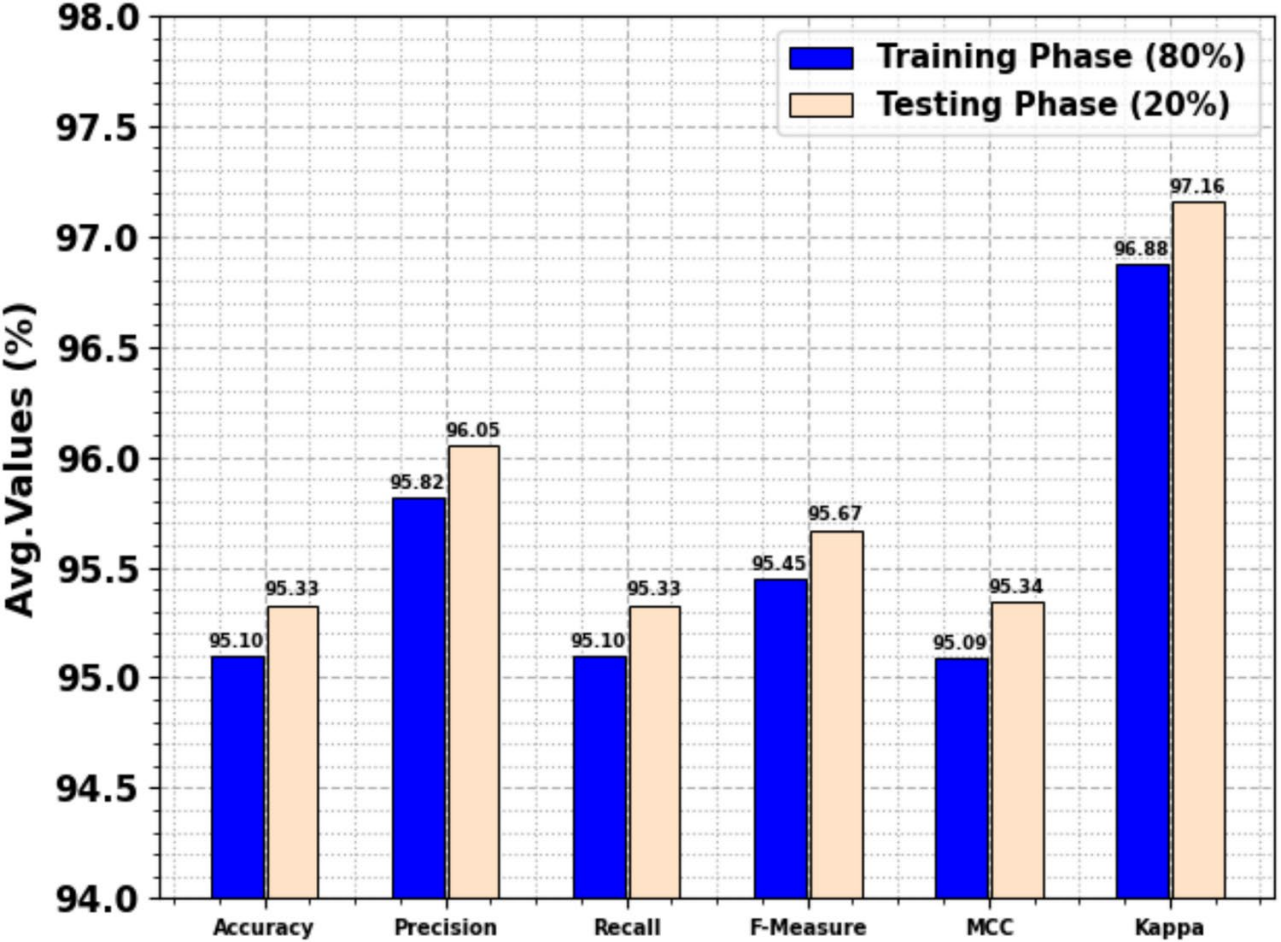
Average of OEMPTER-ISCSO model under 80%TRPH and 20%TSPH.

Table 5 and Fig. 6 depict the text emotion recognition of the OEMPTER-ISCSO approach below 80%TRPH and 20%TSPH. The performance implies that the OEMPTER-ISCSO approach has gained efficient performance. According to 80%TRPH, the OEMPTER-ISCSO approach got average $acc{u}_{y}$, $pre{c}_{n}$, $rec{a}_{l}$, ${F1}_{measure}$, $MCC$, and Kappa of 95.10%, 95.82%, 95.10%, 95.45%, 95.09%, and 96.88%, respectively. Similarly, according to 20%TSPH, the OEMPTER-ISCSO technique achieved average $acc{u}_{y}$, $pre{c}_{n}$, $rec{a}_{l}$, ${F1}_{measure}$, $MCC$, and Kappa of 95.33%, 96.05%, 95.33%, 95.67%, 95.34%, and 97.16%, respectively.

**Table 5:** Text emotion detection of OEMPTER-ISCSO model under 80%TRPH and 20%TSPH.

**Class Labels**	${\varvec{A}}{\varvec{c}}{\varvec{c}}{{\varvec{u}}}_{{\varvec{y}}}$	${\varvec{P}}{\varvec{r}}{\varvec{e}}{{\varvec{c}}}_{{\varvec{n}}}$	${\varvec{R}}{\varvec{e}}{\varvec{c}}{{\varvec{a}}}_{{\varvec{l}}}$	${{\varvec{F}}1}_{{\varvec{m}}{\varvec{e}}{\varvec{a}}{\varvec{s}}{\varvec{u}}{\varvec{r}}{\varvec{e}}}$	${\varvec{M}}{\varvec{C}}{\varvec{C}}$	**Kappa**
**Training Phase (80%)**						
Empty	94.67	94.08	94.67	94.37	94.08	96.75
Sadness	98.48	98.41	98.48	98.45	97.75	98.55
Enthusiasm	94.57	95.08	94.57	94.82	94.59	96.24
Neutral	92.31	94.66	92.31	93.47	93.27	95.01
Worry	85.64	90.81	85.64	88.15	87.82	92.88
Surprise	98.33	97.55	98.33	97.94	97.64	98.86
Fun	97.92	97.28	97.92	97.60	97.32	97.82
Happiness	98.89	98.72	98.89	98.81	98.28	98.92
**Average**	**95.10**	**95.82**	**95.10**	**95.45**	**95.09**	**96.88**
**Testing Phase (20%)**						
Empty	92.59	93.17	92.59	92.88	92.51	93.24
Sadness	98.98	98.48	98.98	98.73	98.17	98.80
Enthusiasm	94.59	95.24	94.59	94.92	94.67	97.42
Neutral	92.78	93.75	92.78	93.26	93.06	98.15
Worry	88.54	94.44	88.54	91.40	91.19	97.73
Surprise	98.69	96.92	98.69	97.80	97.51	96.23
Fun	97.72	97.17	97.72	97.44	97.13	97.71
Happiness	98.73	99.21	98.73	98.97	98.50	98.01
**Average**	**95.33**	**96.05**	**95.33**	**95.67**	**95.34**	**97.16**

**Fig. 7:**
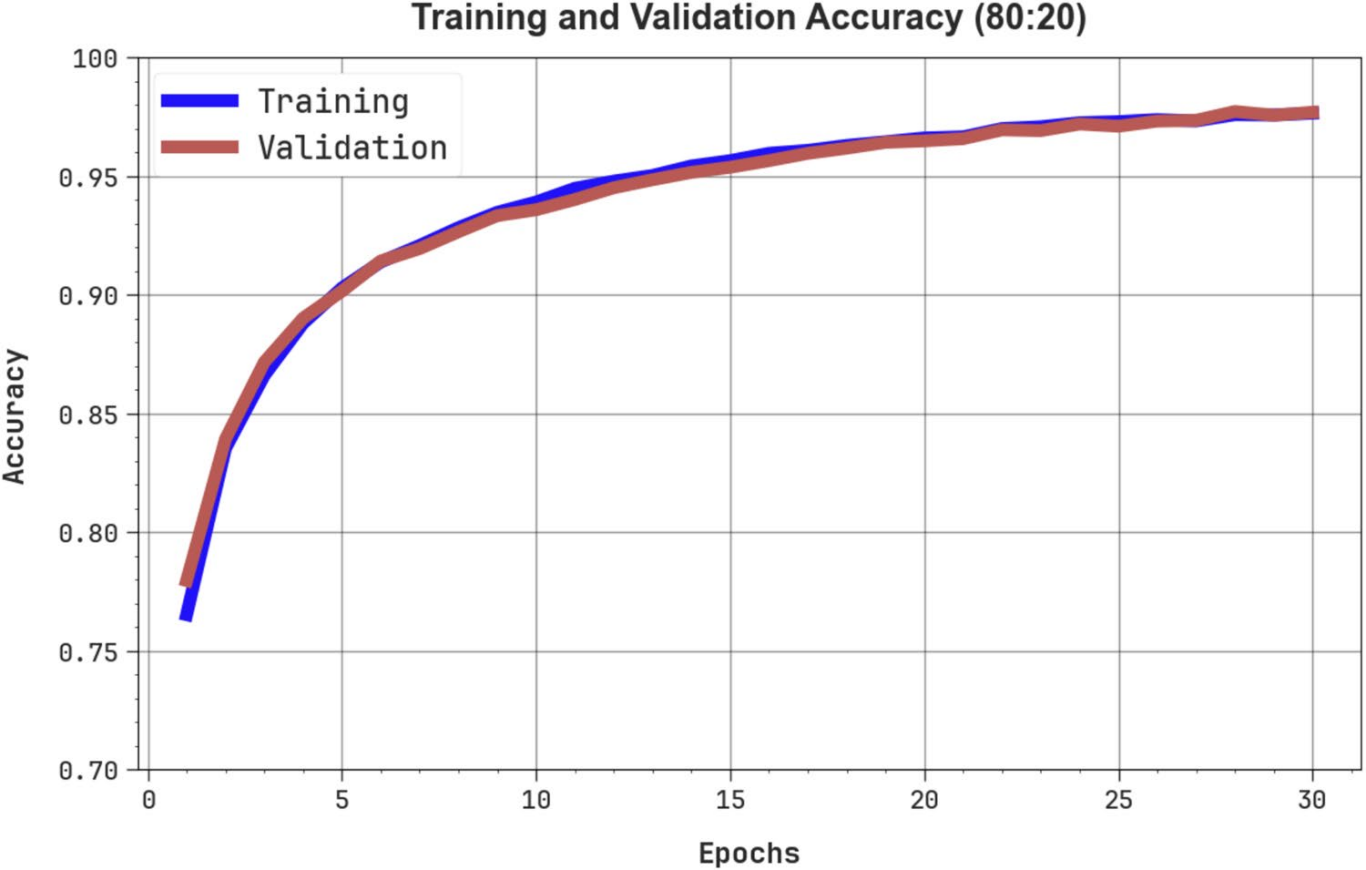
$Acc{u}_{y}$ curve of OEMPTER-ISCSO model under 80%TRPH and 20%TSPH.

In Fig. 7, the training (TRA) $acc{u}_{y}$ and validation (VAL) $acc{u}_{y}$ performances of the OEMPTER-ISCSO model under 80%TRPH and 20%TSPH are shown. The $acc{u}_{y}$ values are calculated through a period of 0–30 epochs. The figure noted that the values of TRA and VAL $acc{u}_{y}$ present an increasing trend, indicating the competency of the OEMPTER-ISCSO method with maximum performance across numerous repetitions. Moreover, the TRA and VAL $acc{u}_{y}$ values remain close throughout the epochs, indicating diminished overfitting and demonstrating the optimal outcome of the OEMPTER-ISCSO method, which ensures reliable calculations on unseen samples.

**Fig. 8:**
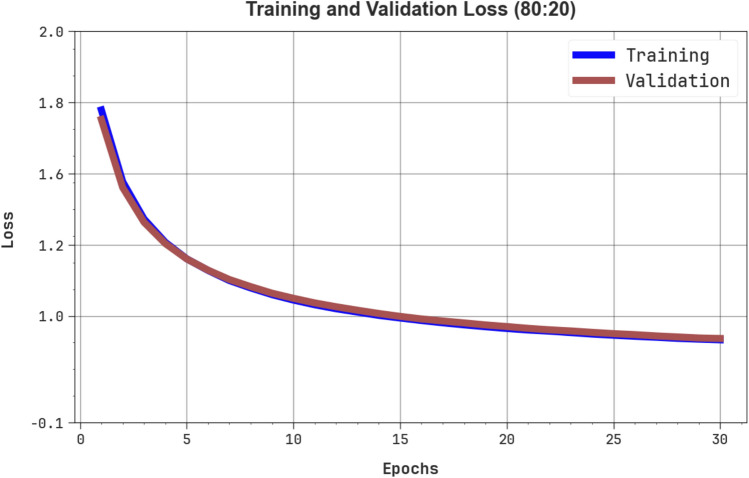
Loss curve of OEMPTER-ISCSO model under 80%TRPH and 20%TSPH.

Fig. 8 demonstrates the TRA loss (TRALOS) and VAL loss (VALLOS) graph of the OEMPTER-ISCSO model under 80%TRPH and 20%TSPH. The loss values are computed across 0 to 30 epochs. The values of TRALOS and VALLOS represent a diminishing tendency, indicating the proficiency of the OEMPTER-ISCSO method in harmonizing a tradeoff between data fitting and generalization. The consecutive decrease in loss and securities values enhanced the outcome of the OEMPTER-ISCSO method and eventually tuned the forecast solutions.

**Fig. 9:**
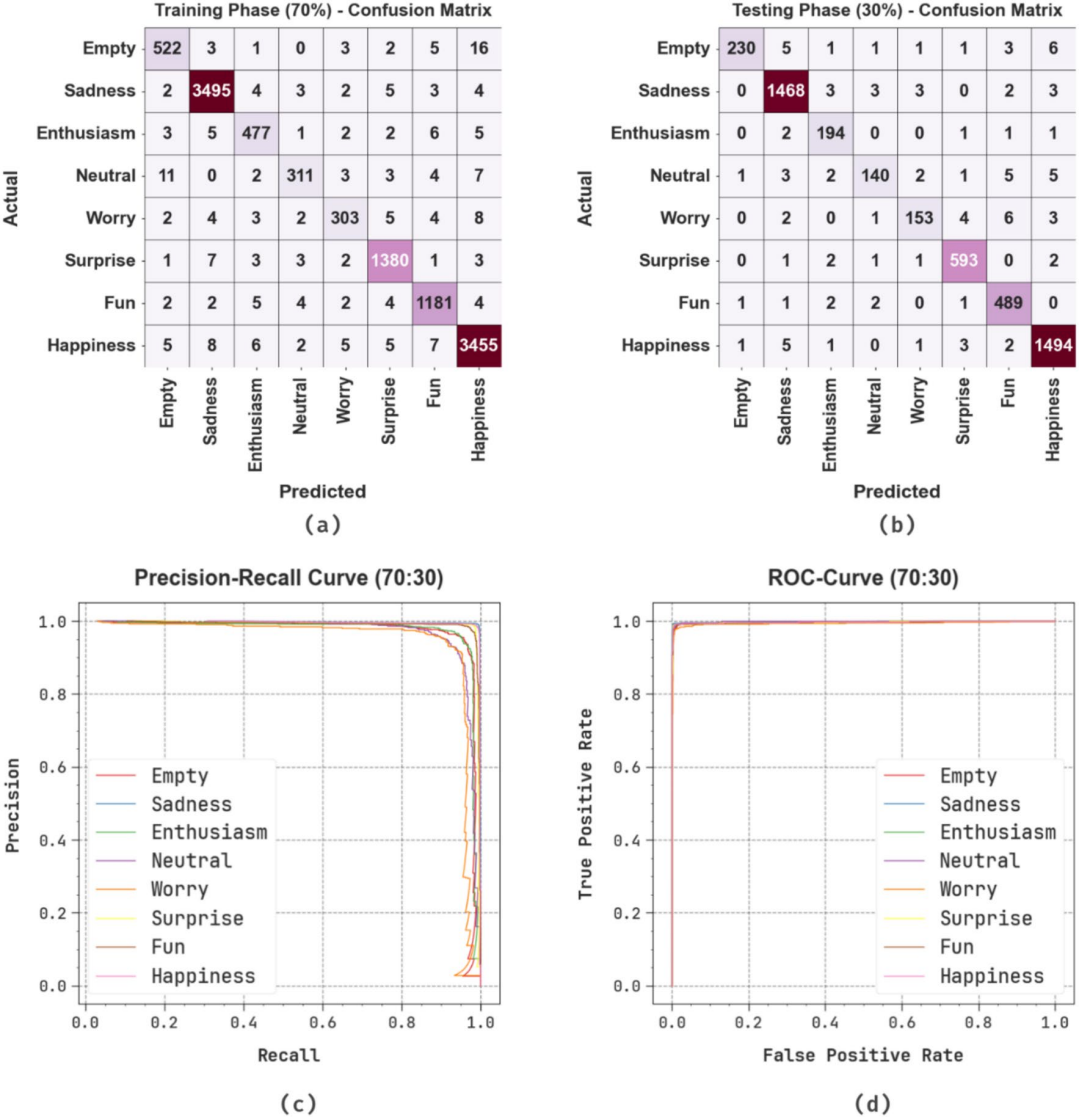
70%TRPH and 30%TSPH of (**a**-**b**) confusion matrices and (**c**-**d**) curves of PR and ROC.

Fig. 9 exhibits the classifier analysis of the OEMPTER-ISCSO technique below 70%TRPH and 30%TSPH. Fig. 9a and Fig. 9b display the confusion matrix, which provides precise classification and identification of all classes. Fig. 9c-9d displays the PR and ROC curves, which show superior performance across all class labels. The TRPH exhibits robust classification performance, with high correct predictions in major classes such as Sadness and Happiness, while the TSPH consistently yields accurate predictions across categories. The PR curve illustrates high precision for most emotions, and the ROC curve demonstrates robust true positive rates, especially for classes such as Sadness, Surprise, Fun, and Happiness, emphasizing the efficiency of the model and its discrimination ability across the dataset.

**Fig. 10:**
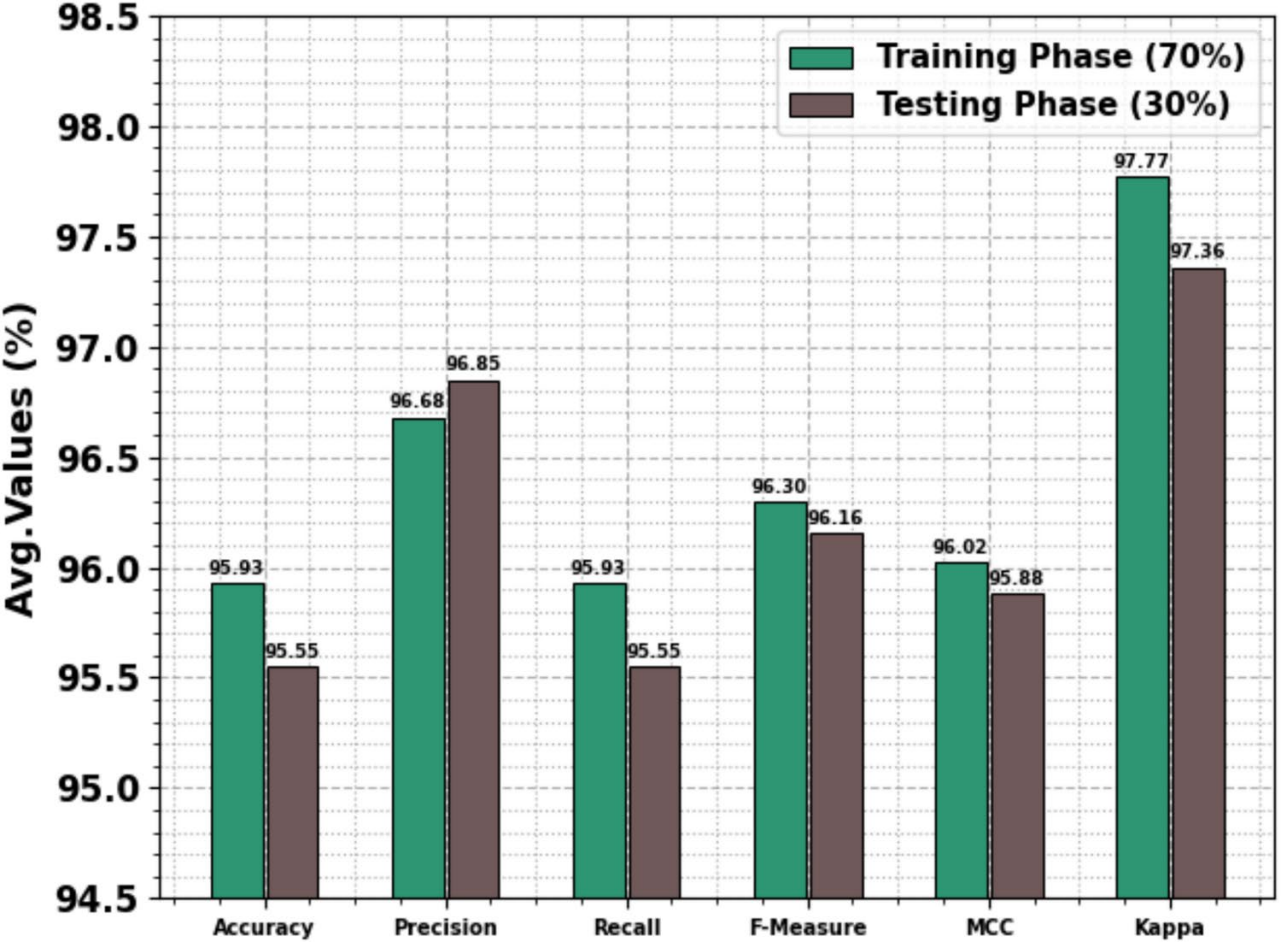
Average of OEMPTER-ISCSO model under 70%TRPH and 30%TSPH.

Table 6 and Fig. 10 depict the text emotion detection of the OEMPTER-ISCSO approach below 70%TRPH and 30%TSPH. The results indicate that the OEMPTER-ISCSO approach has achieved effective performance. According to 70%TRPH, the OEMPTER-ISCSO method attains an average $acc{u}_{y}$, $pre{c}_{n}$, $rec{a}_{l}$, ${F1}_{measure}$, $MCC$, and Kappa of 95.93%, 96.68%, 95.93%, 96.30%, 96.02%, and 97.77%, respectively. Likewise, according to 30%TSPH, the OEMPTER-ISCSO method attains an average $acc{u}_{y}$, $pre{c}_{n}$, $rec{a}_{l}$, ${F1}_{measure}$, $MCC$, and Kappa of 95.55%, 96.85%, 95.55%, 96.16%, 95.88%, and 97.36%, respectively.

**Table 6:** Text emotion detection of OEMPTER-ISCSO model under 70%TRPH and 30%TSPH.

**Class Labels**	${\varvec{A}}{\varvec{c}}{\varvec{c}}{{\varvec{u}}}_{{\varvec{y}}}$	${\varvec{P}}{\varvec{r}}{\varvec{e}}{{\varvec{c}}}_{{\varvec{n}}}$	${\varvec{R}}{\varvec{e}}{\varvec{c}}{{\varvec{a}}}_{{\varvec{l}}}$	${{\varvec{F}}1}_{{\varvec{m}}{\varvec{e}}{\varvec{a}}{\varvec{s}}{\varvec{u}}{\varvec{r}}{\varvec{e}}}$	${\varvec{M}}{\varvec{C}}{\varvec{C}}$	**Kappa**
**Training Phase (70%)**						
Empty	94.57	95.26	94.57	94.91	94.65	95.52
Sadness	99.35	99.18	99.35	99.26	98.93	99.83
Enthusiasm	95.21	95.21	95.21	95.21	94.99	95.86
Neutral	91.20	95.40	91.20	93.25	93.07	97.91
Worry	91.54	94.10	91.54	92.80	92.60	96.83
Surprise	98.57	98.15	98.57	98.36	98.13	98.94
Fun	98.09	97.52	98.09	97.81	97.54	98.15
Happiness	98.91	98.66	98.91	98.78	98.24	99.12
**Average**	**95.93**	**96.68**	**95.93**	**96.30**	**96.02**	**97.77**
**Testing Phase (30%)**						
Empty	92.74	98.71	92.74	95.63	95.46	96.17
Sadness	99.06	98.72	99.06	98.89	98.40	99.28
Enthusiasm	97.49	94.63	97.49	96.04	95.88	96.72
Neutral	88.05	94.59	88.05	91.21	90.98	97.58
Worry	90.53	95.03	90.53	92.73	92.50	93.17
Surprise	98.83	98.18	98.83	98.50	98.29	99.06
Fun	98.59	96.26	98.59	97.41	97.12	97.74
Happiness	99.14	98.68	99.14	98.91	98.42	99.17
**Average**	**95.55**	**96.85**	**95.55**	**96.16**	**95.88**	**97.36**

**Fig. 11:**
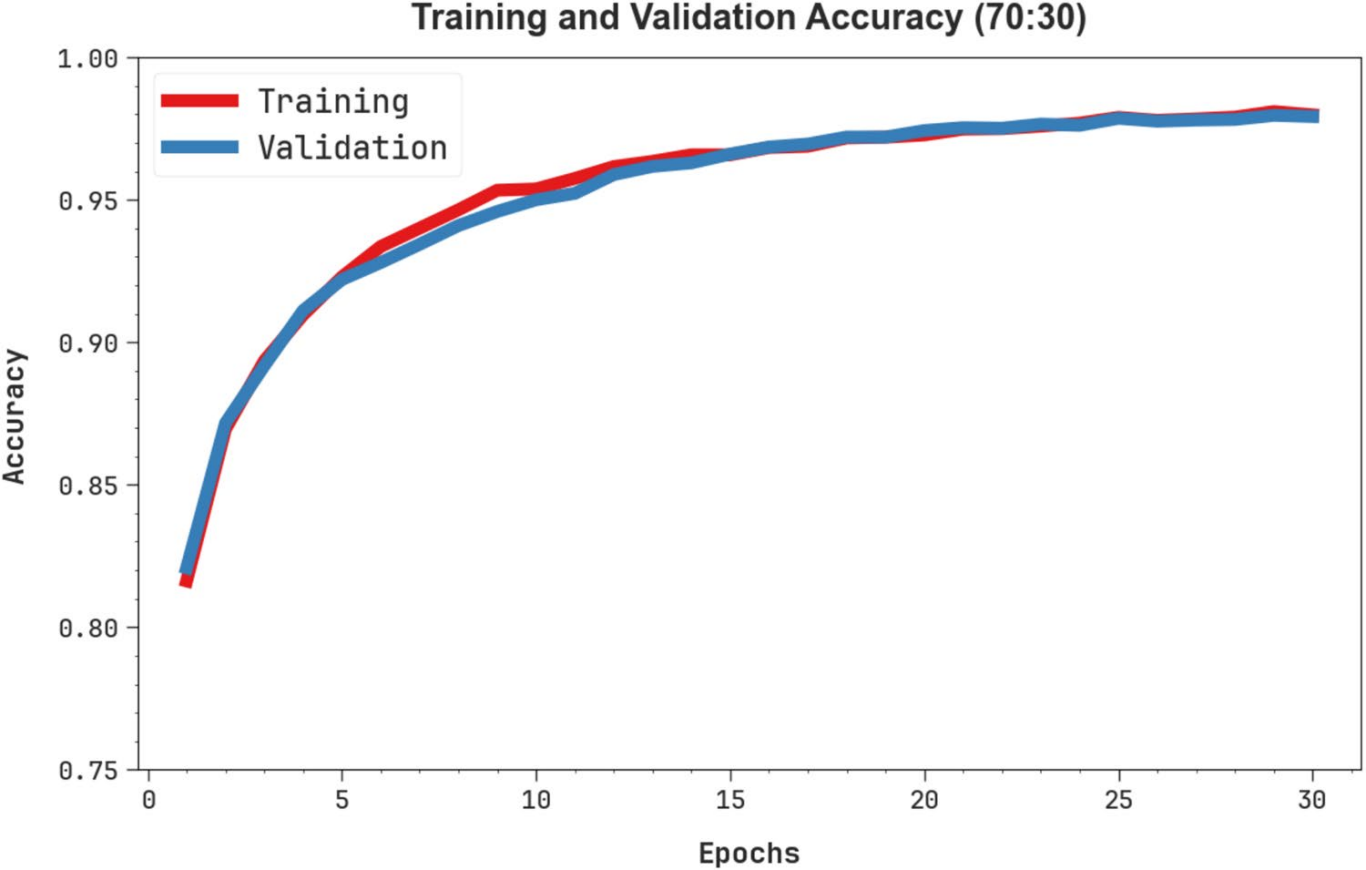
$Acc{u}_{y}$ curve of OEMPTER-ISCSO model under 70%TRPH and 30%TSPH.

Fig. 11 shows the TRA $acc{u}_{y}$ and VAL $acc{u}_{y}$ performances of the OEMPTER-ISCSO methodology below 70%TRPH and 30%TSPH. The $acc{u}_{y}$ values are calculated through a period of 0–30 epochs. The figure underscored that the values of TRA and VAL $acc{u}_{y}$ show a cumulative trend, indicating the proficiency of the OEMPTER-ISCSO technique with enhanced performance through multiple repetitions. Additionally, the TRA and VAL $acc{u}_{y}$ values remain relatively close across the epochs, indicating lesser overfitting and suggesting an improved performance of the OEMPTER-ISCSO technique, which ensures steady predictions on unseen samples.

**Fig. 12:**
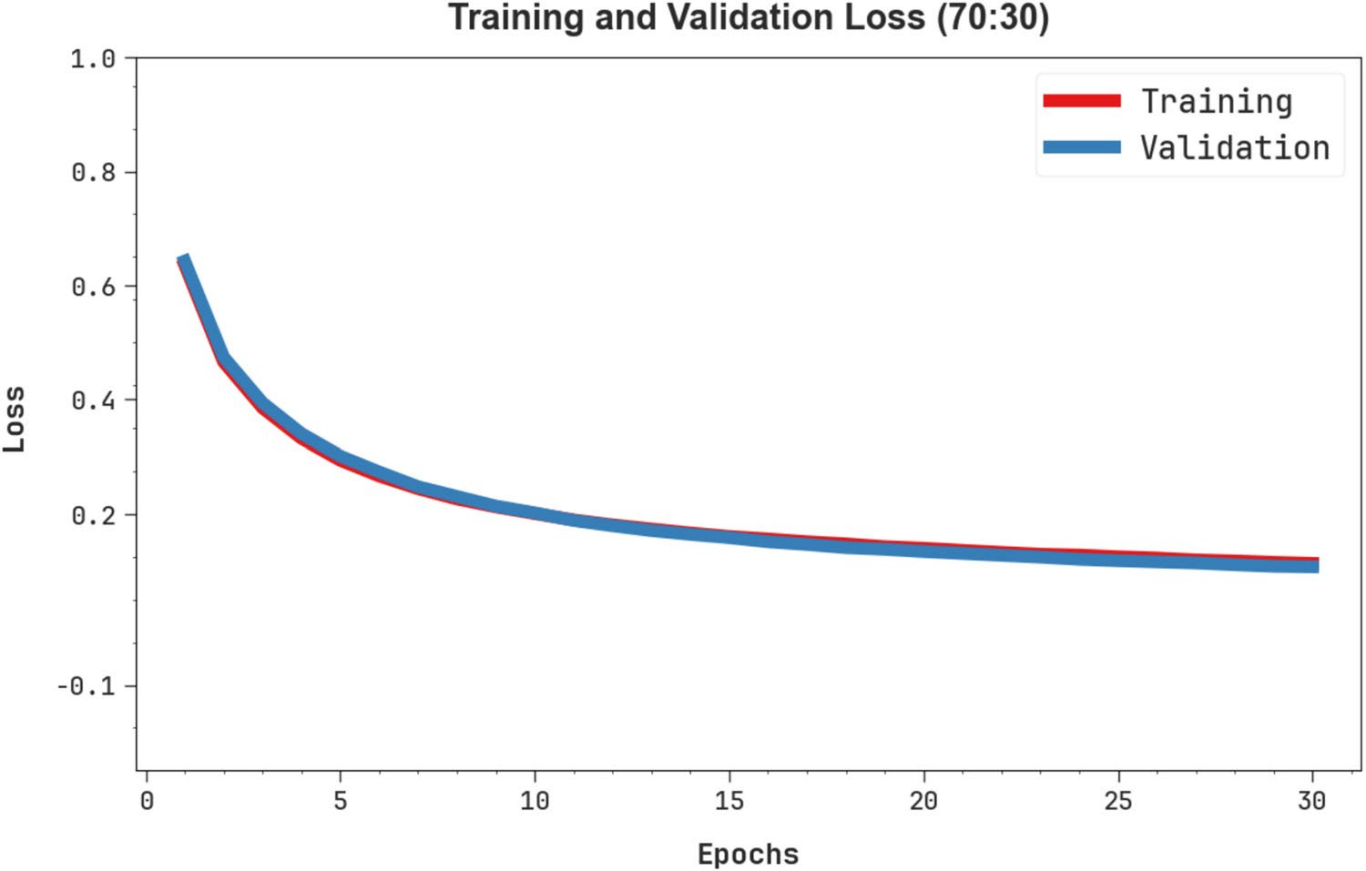
Loss curve of OEMPTER-ISCSO model under 70%TRPH and 30%TSPH.

Fig. 12 presents the TRALOS and VALLOS graphs of the OEMPTER-ISCSO model under 70%TRPH and 30%TSPH. The loss values are computed over a period of 0 to 30 epochs. The values of TRALOS and VALLOS exhibit a reducing trend, which indicates the competency of the OEMPTER-ISCSO approach in balancing the tradeoff between generalization and data fitting. The successive reduction in loss values also ensures the maximum performance of the OEMPTER-ISCSO approach and tunes the prediction results over time.

**Fig. 13:**
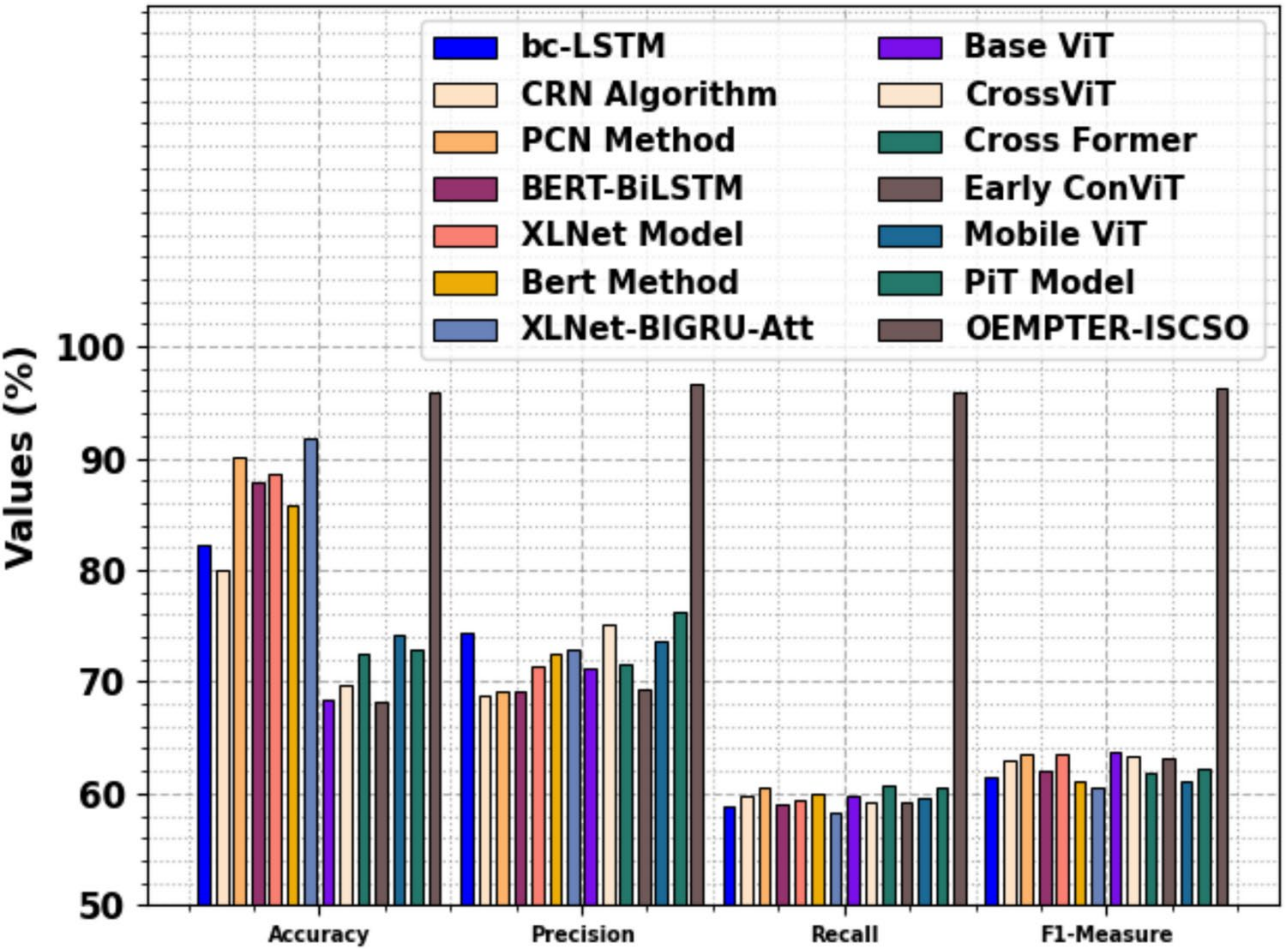
Comparative analysis of OEMPTER-ISCSO model with existing approaches.

Table 7 and Fig. 13 inspect the comparative study of the OEMPTER-ISCSO method with existing methodologies^20,21,50^. The performances indicated that the bc-LSTM, CRN, PCN, BERT-BiLSTM, XLNet, Bert, XLNet-BIGRU-Att, Base ViT, CrossViT, Cross Former, Early Convolutional ViT (Early ConViT), Mobile ViT, and Pooling‑based Vision Transformer (PiT) techniques have reached poorer performance. At the same time, the proposed OEMPTER-ISCSO approach has respective effective values of $acc{u}_{y}$, $pre{c}_{n}$, $rec{a}_{l},$ and ${F1}_{measure}$ of 95.93%, 96.68%, 95.93%, and 96.30%, correspondingly.

**Table 7:** Comparative study of OEMPTER-ISCSO model with existing approaches^20,21,50^.

**Approach**	${\varvec{A}}{\varvec{c}}{\varvec{c}}{{\varvec{u}}}_{{\varvec{y}}}$	${\varvec{P}}{\varvec{r}}{\varvec{e}}{{\varvec{c}}}_{{\varvec{n}}}$	${\varvec{R}}{\varvec{e}}{\varvec{c}}{{\varvec{a}}}_{{\varvec{l}}}$	${{\varvec{F}}1}_{{\varvec{m}}{\varvec{e}}{\varvec{a}}{\varvec{s}}{\varvec{u}}{\varvec{r}}{\varvec{e}}}$
bc-LSTM	82.33	74.38	58.90	61.45
CRN Algorithm	79.90	68.68	59.74	63.05
PCN Method	90.11	69.06	60.52	63.53
BERT-BiLSTM	87.92	69.09	59.01	62.02
XLNet Model	88.64	71.46	59.40	63.51
Bert Method	85.86	72.58	60.00	61.03
XLNet-BIGRU-Att	91.71	72.93	58.34	60.52
Base ViT	68.34	71.13	59.78	63.76
CrossViT	69.74	75.08	59.19	63.34
Cross Former	72.47	71.51	60.64	61.85
Early ConViT	68.24	69.40	59.22	63.18
Mobile ViT	74.28	73.69	59.52	61.09
PiT Model	72.84	76.27	60.47	62.26
OEMPTER-ISCSO	95.93	96.68	95.93	96.30

**Fig. 14:**
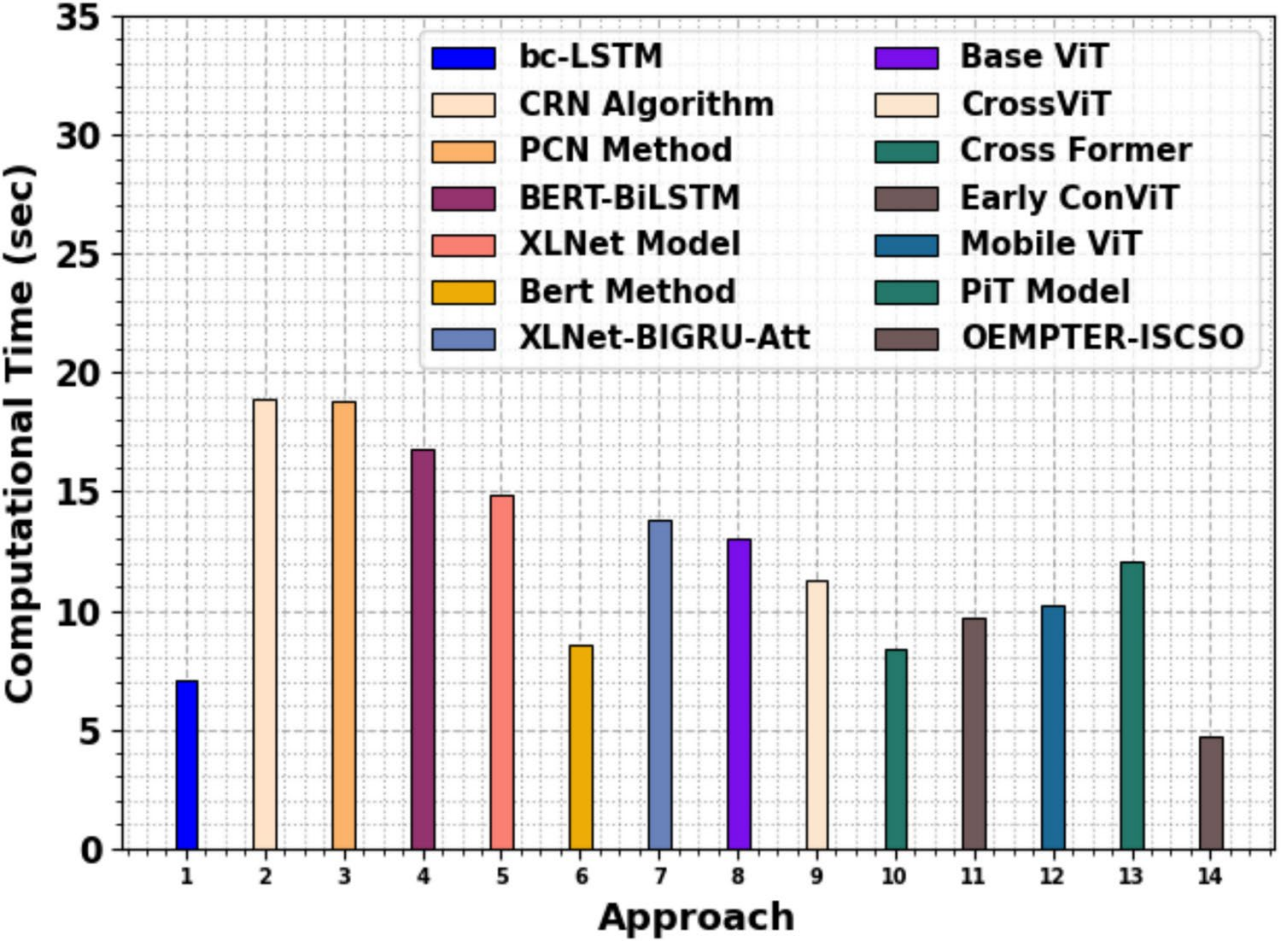
CT outcome of OEMPTER-ISCSO technique with existing models.

The comparative analysis of the OEMPTER-ISCSO technique is presented in terms of computation time (CT) in Table 8 and Fig. 14. The results indicate that the OEMPTER-ISCSO model achieves a superior performance. The OEMPTER-ISCSO approach presents minimal CT of 04.71sec while the bc-LSTM, CRN, PCN, BERT-BiLSTM, XLNet, Bert, XLNet-BIGRU-Att, Base ViT, CrossViT, Cross Former, Early ConViT, Mobile ViT, and PiT models attain improved CT values of 07.10sec, 18.89sec, 18.82sec, 16.78sec, 14.84sec, 08.56sec, 13.83sec, 13.05sec, 11.30sec, 8.406sec, 9.708sec, 10.18sec, 12.05sec, respectively.

**Table 8:** CT outcome of OEMPTER-ISCSO technique with existing models.

**Approach**	**CT (sec)**
bc-LSTM	07.10
CRN Algorithm	18.89
PCN Method	18.82
BERT-BiLSTM	16.78
XLNet Model	14.84
Bert Method	08.56
XLNet-BIGRU-Att	13.83
Base ViT	13.05
CrossViT	11.30
Cross Former	8.406
Early ConViT	9.708
Mobile ViT	10.18
PiT Model	12.05
OEMPTER-ISCSO	04.71

Table 9 demonstrates the ablation study of the OEMPTER-ISCSO methodology. The outputs show that applying ISCO to each model Deep Belief Network (DBN), Elman Neural Network (ELNN), and Temporal CNN (TCNN) consistently enhance performance across all metrics, including $acc{u}_{y}$, $pre{c}_{n}$, $rec{a}_{l},$ and ${F1}_{measure}$. For instance, the TCNN model with ISCO attains the highest $acc{u}_{y}$ of 95.84% and an ${F1}_{measure}$ of 65.45%, compared to 95.02% and 64.87% without ISCO, highlighting the efficiency of the ISCO method in improving generalization and fine-tuning. The fusion model, without hyperparameter tuning, attains lower than individual ISCO-optimized models, emphasizing the significance of ISCO in optimizing model parameters and contributing significantly to enhanced emotion recognition performance.

**Table 9:** Comparative performance evaluation of the OEMPTER-ISCSO methodology through ablation study against existing techniques.

**Methodology**	${\varvec{A}}{\varvec{c}}{\varvec{c}}{{\varvec{u}}}_{{\varvec{y}}}$	${\varvec{P}}{\varvec{r}}{\varvec{e}}{{\varvec{c}}}_{{\varvec{n}}}$	${\varvec{R}}{\varvec{e}}{\varvec{c}}{{\varvec{a}}}_{{\varvec{l}}}$	${{\varvec{F}}1}_{{\varvec{m}}{\varvec{e}}{\varvec{a}}{\varvec{s}}{\varvec{u}}{\varvec{r}}{\varvec{e}}}$
DBN (without ISCO)	92.22	78.31	57.56	61.96
DBN (with ISCO)	92.75	78.86	58.41	62.69
ENN (without ISCO)	93.61	79.39	59.06	63.52
ENN (with ISCO)	94.38	79.95	59.84	64.02
TCNN (without ISCO)	95.02	80.79	60.63	64.87
TCNN (with ISCO)	95.84	81.44	61.19	65.45
OEMPTER-ISCSO (Fusion Model with hyperparameter selection)	95.93	96.68	95.93	96.30

## Conclusion

This manuscript presents an OEMPTER-ISCSO method. Initially, the text pre-processing stage involves multiple levels to normalize and clean an input text. Then, the FastText method is employed for the word embedding process, transforming words into numerical vector representations. An ensemble of three classifiers, EDBN, ELNN, and ITCN methods, is used for textual emotion detection. Additionally, the ISCO model-based hyperparameter selection process is executed to optimize the detection outcomes of the ensemble models. The experimentation of the OEMPTER-ISCSO technique is accomplished using emotion detection from a text dataset. The performance validation of the OEMPTER-ISCSO technique demonstrated a superior accuracy value of 95.84% over existing models. The limitations of the OEMPTER-ISCSO technique include reliance on specific datasets that may not fully represent the diverse range of real-world scenarios, potentially restricting the generalizability of the findings. Moreover, the proposed models’ computational complexity and resource-intensive behaviour may affect their deployment in resource-constrained environments. The study also faces challenges in handling noisy and incomplete data, which could impact the accuracy of predictions. Furthermore, the real-time performance of the system under varying conditions needs additional optimization to ensure scalability. Future work should focus on improving the robustness of the model by integrating more diverse datasets and optimizing computational efficiency for real-time applications. Additionally, it could extend its practical utility by incorporating hybrid approaches and exploring the model’s applicability in other domains, such as healthcare and industrial automation.

## Data Availability

The data supporting this study’s findings are openly available in the Kaggle repository at https://www.kaggle.com/datasets/pashupatigupta/emotion-detection-from-text, reference number^49^.
